# Sphingolipid subtypes differentially control proinsulin processing and systemic glucose homeostasis

**DOI:** 10.1038/s41556-022-01027-2

**Published:** 2022-12-21

**Authors:** Kerstin Griess, Michael Rieck, Nadine Müller, Gergely Karsai, Sonja Hartwig, Angela Pelligra, Robert Hardt, Caroline Schlegel, Jennifer Kuboth, Celina Uhlemeyer, Sandra Trenkamp, Kay Jeruschke, Jürgen Weiss, Leon Peifer-Weiss, Weiwei Xu, Sandra Cames, Xiaoyan Yi, Miriam Cnop, Mathias Beller, Holger Stark, Arun Kumar Kondadi, Andreas S. Reichert, Daniel Markgraf, Marianne Wammers, Dieter Häussinger, Oliver Kuss, Stefan Lehr, Decio Eizirik, Heiko Lickert, Eckhard Lammert, Michael Roden, Dominic Winter, Hadi Al-Hasani, Doris Höglinger, Thorsten Hornemann, Jens C. Brüning, Bengt-Frederik Belgardt

**Affiliations:** 1 Institute for Vascular and Islet Cell Biology, German Diabetes Center (DDZ), Leibniz Center for Diabetes Research at Heinrich Heine University Düsseldorf, Düsseldorf, Germany; 2 German Center for Diabetes Research (DZD e.V.), Neuherberg, Germany; 3 Center for Integrative Human Physiology, University of Zürich, Zürich, Switzerland; 4 Institute for Clinical Chemistry, University Hospital, Zürich, Switzerland; 5 Institute for Clinical Biochemistry and Pathobiochemistry, German Diabetes Center, Leibniz Center for Diabetes Research at Heinrich Heine University Düsseldorf, Düsseldorf, Germany; 6 Institute for Biochemistry and Molecular Biology, Medical Faculty, Rheinische Friedrich-Wilhelms-University Bonn, Bonn, Germany; 7 Institute for Clinical Diabetology, German Diabetes Center, Leibniz Center for Diabetes Research at Heinrich Heine University Düsseldorf, Düsseldorf, Germany; 8 Institute of Diabetes and Regeneration Research, Helmholtz Center Munich, Neuherberg, Germany; 9 ULB Center for Diabetes Research, Medical Faculty, Université Libre De Bruxelles, Brussels, Belgium; 10 Division of Endocrinology, Erasmus Hospital, Université Libre de Bruxelles, Brussels, Belgium; 11 Institute for Mathematical Modeling of Biological Systems and Systems Biology of Lipid Metabolism, Heinrich Heine University Düsseldorf, Düsseldorf, Germany; 12 Institute of Pharmaceutical and Medicinal Chemistry, Heinrich Heine University Düsseldorf, Düsseldorf, Germany; 13 Institute of Biochemistry and Molecular Biology I, Medical Faculty and University Hospital Düsseldorf, Heinrich Heine University Düsseldorf, Düsseldorf, Germany; 14 Department of Gastroenterology, Hepatology and Infectious Diseases, Heinrich Heine University Düsseldorf, Düsseldorf, Germany; 15 Institute for Biometrics and Epidemiology, German Diabetes Center (DDZ), Leibniz Center for Diabetes Research at Heinrich Heine University Düsseldorf, Düsseldorf, Germany; 16 Centre for Health and Society, Medical Faculty, Heinrich Heine University Düsseldorf, Düsseldorf, Germany; 17 Welbio, Medical Faculty, Université Libre de Bruxelles, Brussels, Belgium; 18 Department of Medicine, Technical University of Munich, Munich, Germany; 19 Institute of Metabolic Physiology, Heinrich Heine University Düsseldorf, Düsseldorf, Germany; 20 Department of Endocrinology and Diabetology, Medical Faculty and University Hospital Düsseldorf, Heinrich Heine University Düsseldorf, Düsseldorf, Germany; 21 Medical Faculty, Heinrich Heine University Düsseldorf, Düsseldorf, Germany; 22 Heidelberg University Biochemistry Center, Heidelberg, Germany; 23 Department of Neuronal Control of Metabolism, Max Planck Institute for Metabolism Research, Cologne, Germany; 24 Policlinic for Endocrinology, Diabetes and Preventive Medicine (PEDP), University Hospital Cologne, Cologne, Germany; 25 Excellence Cluster on Cellular Stress Responses in Aging-Associated Diseases (CECAD), University of Cologne, Cologne, Germany; 26 Center for Molecular Medicine Cologne (CMMC), University of Cologne, Cologne, Germany

**Keywords:** Type 2 diabetes, Mechanisms of disease, Molecular biology

## Abstract

Impaired proinsulin-to-insulin processing in pancreatic β-cells is a key defective step in both type 1 diabetes and type 2 diabetes (T2D) (refs. ^1^^,^^2^), but the mechanisms involved remain to be defined. Altered metabolism of sphingolipids (SLs) has been linked to development of obesity, type 1 diabetes and T2D (refs. ^3–8^); nonetheless, the role of specific SL species in β-cell function and demise is unclear. Here we define the lipid signature of T2D-associated β-cell failure, including an imbalance of specific very-long-chain SLs and long-chain SLs. β-cell-specific ablation of CerS2, the enzyme necessary for generation of very-long-chain SLs, selectively reduces insulin content, impairs insulin secretion and disturbs systemic glucose tolerance in multiple complementary models. In contrast, ablation of long-chain-SL-synthesizing enzymes has no effect on insulin content. By quantitatively defining the SL–protein interactome, we reveal that CerS2 ablation affects SL binding to several endoplasmic reticulum–Golgi transport proteins, including Tmed2, which we define as an endogenous regulator of the essential proinsulin processing enzyme Pcsk1. Our study uncovers roles for specific SL subtypes and SL-binding proteins in β-cell function and T2D-associated β-cell failure.

## Abstract (web summary)

Griess et al. determine the lipid signatures of pancreatic islets in type 2 diabetes models. They define a lipid environment linking the endoplasmic reticulum–Golgi transport protein Tmed2 to the proinsulin processing enzyme Pcsk1 to ensure insulin production.

## Main

Proinsulin processing is impaired in patients with type 2 diabetes (T2D), but messenger RNA expression of the evolutionary conserved enzyme proprotein convertase subtilisin/kexin type 1 (Pcsk1) (refs. ^9,10^) and other proinsulin processing enzymes appears to be unaffected in pancreatic islets from T2D donors^11^, indicating that post-transcriptional pathomechanisms may impact insulin processing. With hyperlipidaemia representing a hallmark of both obesity and T2D, elevated circulating concentrations of fatty acids are potential ‘lipotoxic’ drivers of cellular impairment in metabolic disease, although mechanistic insights into lipotoxicity in vivo are still limited^3,12–16^. Sphingolipids (SLs) constitute a complex class of lipids, with multiple functions in virtually all cell types and membranes^3,4^. SLs are synthesized by condensation of palmitate with serine to generate a sphingoid backbone. Afterwards, six different ceramide synthases (CerS1-6) link a second fatty acid of specific chain length to this backbone, generating ceramides (Cer). This second fatty acid (‘side-chain’) can vary between 14 and more than 30 carbon atoms. Cer can be further modified at the C1 hydroxyl residue in the sphingosine backbone by sugar moieties or phosphatidylcholine, leading to the generation of hexosylceramide (HexCer) and sphingomyelin (SM) species, respectively, and hence hundreds of SLs with probably different biological functions.

**Fig. 1:**
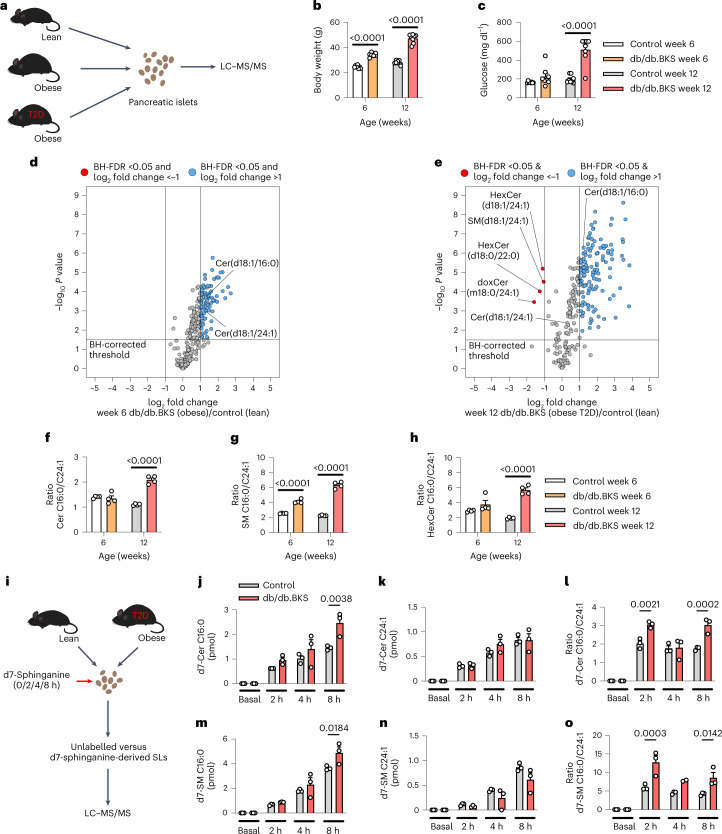
A lipid signature of T2D-associated β-cell failure. **a**, Experimental design for lipidomics of T2D islets. **b**,**c**, Body weight and blood glucose levels of control and db/db.BKS mice at weeks 6 and 12 (*n* = 8 control versus 8 db/db.BKS mice per age). **d**,**e**, Volcano plot showing log_2_ fold change of lipids in islets between lean control and obese but normoglycaemic db/db.BKS mice at 6 weeks of age (**d**) or in islets of lean control and obese and diabetic db/db.BKS mice at 12 weeks of age (**e**) plotted against the −log_10_ *P* value of a two-sided equal variance *t*-test. Log_2_ fold change >1 and BH-FDR <0.05 was used as cut-off for significance (*n* = 4 control versus 4 db/db.BKS islet replicates per age). Only lipids detected in all samples were used for calculation. Full dataset can be found in Supplementary Tables 1 and 2. **f**–**h**, Ratio of C16:0/C24:1 ceramides (**f**), SM (**g**) and hexosylceramides (**h**) in control and db/db.BKS islets at the age of 6 and 12 weeks (*n* = 4 control versus 4 db/db.BKS islet replicates per age; same dataset as in **d** and **e**). **i**, Experimental design for investigating de novo sphingolipogenesis in islets from 12-week-old male db/db.BKS and control mice using d7-sphinganine. **j**,**k**, Comparison of de novo synthesized d7-C16:0 (**j**) and d7-C24:1(**k**) ceramides in islets from db/db.BKS and control mice after 0, 2, 4 and 8 h of pulsing with d7-sphinganine. **l**, Ratio of de novo synthesized d7-C16:0/d7-C24:1 ceramides in islets from db/db.BKS and control mice after 0, 2, 4 and 8 h of pulsing with d7-sphinganine. **m**,**n**, Comparison of de novo synthesized d7-C16:0 (m) and d7-C24:1 (**n**) SMs in islets from db/db.BKS and control mice after 0, 2, 4 and 8 h of pulsing with d7-sphinganine. **o**, Ratio of de novo synthesized d7-C16:0/d7-C24:1 SMs in islets from db/db.BKS and control mice after 0, 2, 4 and 8 h of pulsing with d7-sphinganine. For **j**–**o**, *n* = 3 control versus 3 db/db.BKS islet replicates. Statistical analysis in **b**, **c** and **f**–**h** was performed using two-way ANOVA with uncorrected Fisher’s least significant difference test. Two-way ANOVA with Sidak’s multiple comparisons test was used in **j**–**o**. *P* values are stated in each figure. Data points in **b** and **c** indicate individual mice. Data points in **f**–**h** represent islet replicates; one replicate equals 65 islets picked from one to two pools of islets from four to eight individual mice, respectively. Data points in **j**–**o** represent islet replicates; one replicate equals 60 islets picked from one islet pool from nine to ten individual mice, respectively. Bar graphs represent mean ± s.e.m. Source numerical data are available in source data. Source data

**Extended Data Fig. 1:**
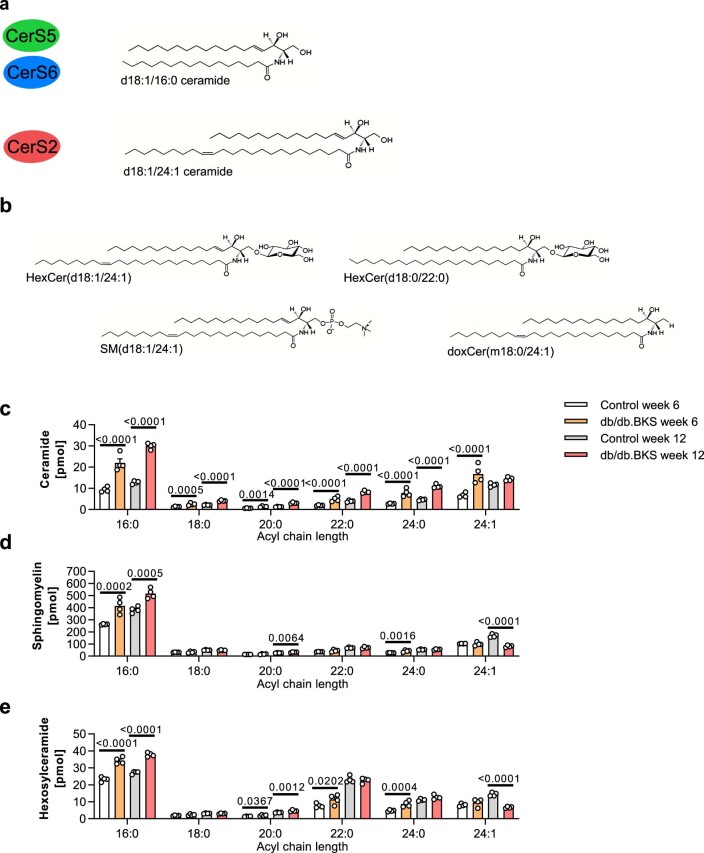
Additional data of islet lipidomics from a T2D model. **a**, Graphical overview of CerS2, CerS5 and CerS6 and their major ceramide products, respectively. **b**, Structures of all four SLs with significantly reduced abundance identified in (Fig. 1e). **c-e**, Comparison of Ceramide (c), sphingomyelin (d) and hexosylceramide (e) species of different side chain lengths in islets of lean control mice, obese and normoglycemic and obese type 2 diabetic mice (*n* = 4 control vs. 4 db/db.BKS islet replicates per age; same dataset as in Fig. 1d-h). Statistical analysis on selected lipid species was performed using two-way ANOVA with uncorrected Fisher’s LSD test. Data points in (c-e) represent islet replicates. One replicate equals 65 islets picked from 1-2 pools of islets from 4-8 individual mice, respectively. Bar graphs represent means + s.e.m. To ensure readability, only *p*-values for selected comparisons are stated in each figure. Source numerical data are available in source data. Source data

**Extended Data Fig. 2:**
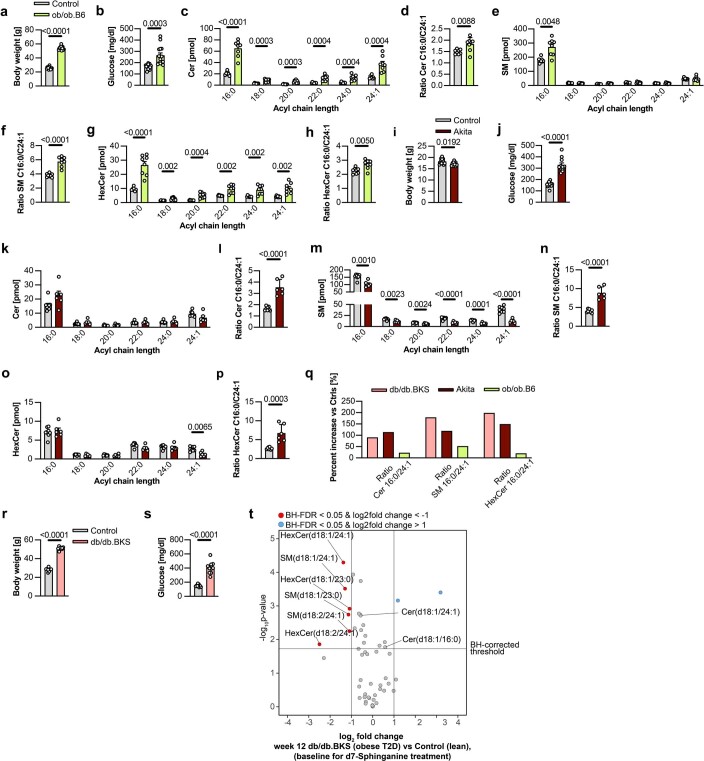
The islet sphingolipidome of Akita and ob/ob.B6 mice. **a**, **b**, Body weight (a) and blood glucose levels (b) of 13 male control and 14 ob/ob.B6 mice at week 12. **c-h**, Ceramide (c), sphingomyelin (e) and hexosylceramide (g) species and ratios of C16:0/C24:1 ceramides (d), sphingomyelins (f) and hexosylceramides (h) in islets of 8 male control and 8 ob/ob.B6 mice at week 12. **i**, **j**, Body weight (i) and blood glucose levels (j) of 12 female control and 11 Akita mice at week 7. **k-p**, Sphingolipid measurements as described in (c-h) in islets of 8 female control and 6 Akita mice at week 7. **q**, Percent increase in the C16:0/C24:1 ratio of specific sphingolipids in islets of male db/db.BKS, female Akita and male ob/ob mice to their respective controls (calculations based on lipidomics analyses shown in Fig. 1 and Extended Data Fig. 1). **r, s**, Body weight (r) and blood glucose levels (s) of 9 male control and 10 db/db.BKS mice at week 12 which were used for analyses shown in Fig. 1j-o. **t**, Volcano plot showing log_2_ fold change of baseline lipid measurements (shown in Fig. 1j-o) plotted against the –log_10_ p-value of a two-sided equal variance *t*-test. Cutoffs for significance as in Fig. 1d, e (*n* = 3 control vs. 3 db/db.BKS islet replicates). Only lipids detected in all samples were used for calculation. Statistics were performed using a two-sided Student’s *t*-test in (a, b, d, f, h-j, l, n, p, r, s) and multiple two-sided *t*-tests with Holm-Sidak correction in (c, e, g, k, m, o). Data points in (a-p, r, s) represent individual mice. Bar graphs in (a-p, r, s) represent means + s.e.m. Bar graphs in (q) represent values based on mean calculations of the respective groups presented in Fig. 1 and in this Figure. Source numerical data are available in source data. Source data

As it is unknown whether β-cell demise is linked to alterations of SL levels, we initially aimed to define the sphingolipidome of pancreatic β-cells during development of T2D. To this end, we performed untargeted lipidomics of islets from a mouse model for obesity and T2D (db/db.BKS mice) and control animals at 6 weeks and 12 weeks of age. This allowed us to differentiate between the molecular effects of obesity-associated insulin hypersecretion of β-cells (db/db.BKS mice at 6 weeks of age) and T2D-associated β-cell failure (db/db.BKS mice at 12 weeks of age) on the same genetic background^17,18^ (Fig. 1a–c and Supplementary Tables 1 and 2). Many lipid species were found to be upregulated, but no lipid species were decreased in islets from obese (normoglycaemic) mice (db/db.BKS mice at 6 weeks, Fig. 1d). In islets from obese diabetic mice (db/db.BKS mice at 12 weeks), a further shift towards upregulation was observed for many lipid species, including several long-chain SLs (LSLs) such as d18:1/16:0 ceramide (further denoted as C16:0 Cer), which can be synthesized by either CerS5 (ref. ^19^) or CerS6 (ref. ^5^) (Fig. 1e and Extended Data Fig. 1a). Notably, four lipid species were significantly downregulated in islets from diabetic mice (Fig. 1e). These four lipids are very-long-chain SLs (VLSLs) that are generated by ceramide synthase 2 (CerS2) during de novo synthesis^20,21^ (Extended Data Fig. 1b), and three of these four had desaturated side-chains^22^. Furthermore, the ratios of C16:0/C24:1 Cer and C16:0/C24:1 HexCer were significantly increased in islets from obese diabetic mice, but not from obese normoglycaemic mice (Fig. 1f–h and Extended Data Fig. 1c–e). A drastic increase in C16:0/24:1 Cer/SM/HexCer ratios was also observed in islets of lean but diabetic Akita mice^23^ but less so in ob/ob.B6 mice, which show massive obesity but retain near-normal glycaemic control^24^ (Extended Data Fig. 2a–q and Supplementary Tables 3 and 4). Interestingly, all detected CerS2-derived Cer and HexCer were upregulated in ob/ob.B6 islets (Extended Data Fig. 2c,g). To determine whether the observed differences in the diabetic db/db.BKS islet sphingolipidome are due to changes in CerS-mediated de novo sphingolipogenesis, we treated islets from control and diabetic db/db.BKS mice with a deuterated Cer precursor (d7-sphinganine) for different time periods. De novo synthesis of CerS6-dependent ceramides and SMs was significantly increased in islets of diabetic mice, also reflected in elevated C16:0/C24:1 Cer and SM ratios (Fig. 1i–o, Extended Data Fig. 2r,s and Supplementary Tables 5 and 6). At baseline, several CerS2-derived VLSLs were again found to be decreased in db/db.BKS islets (Extended Data Fig. 2t). These observations reveal an imbalance of several specific CerS2 generated VLSLs and CerS5/6 generated LSLs as an obesity-independent lipid signature of β-cell failure and overt T2D, at least in part through altered SL synthesis.

**Fig. 2:**
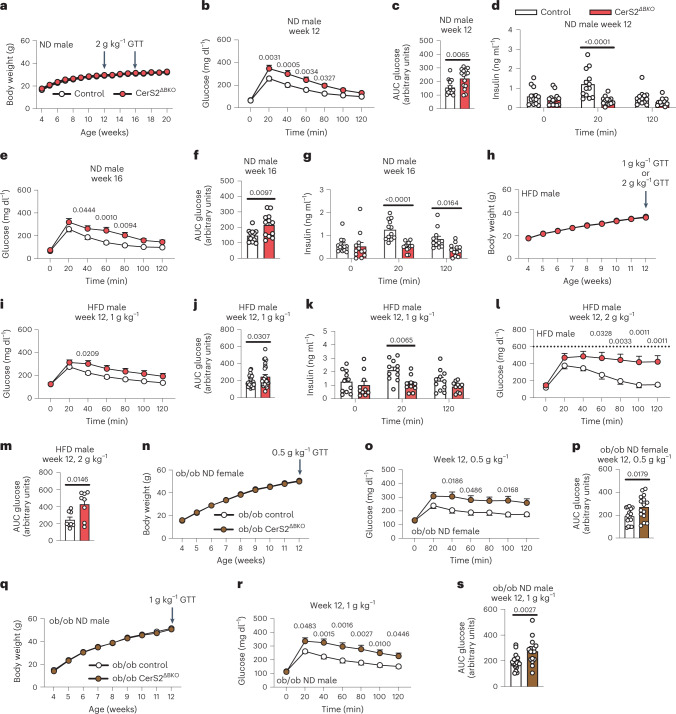
Impaired GSIS and glucose tolerance in CerS2^ΔBKO^ mice. **a**, Body weight of male control and CerS2^ΔBKO^ mice fed ND (*n* = 16-17 control versus 16-17 CerS2^ΔBKO^ mice). **b**, Glucose levels during intra-peritoneal GTT in ND-fed control and CerS2^ΔBKO^ mice at week 12 (*n* = 16 control versus 14 CerS2^ΔBKO^ mice). **c**, Area under the curve (AUC) for glucose levels depicted in **b**. **d**, Plasma insulin levels before injection, 20 min and 120 min after glucose injection in GTT depicted in **b** (*n* = 13 control versus 13 CerS2^ΔBKO^ mice). **e**, Glucose levels during GTT in ND-fed control and CerS2^ΔBKO^ mice at week 16 (*n* = 12 control versus 12 CerS2^ΔBKO^ mice). **f**, AUC for GTT depicted in **e**. **g**, Plasma insulin levels before injection, 20 min and 120 min after glucose injection in GTT depicted in **e** (*n* = 13 control versus 13 CerS2^ΔBKO^ mice). **h**, Body weight of male HFD-fed control and CerS2^ΔBKO^ mice (*n* = 33 control versus 30-32 CerS2^ΔBKO^ mice). **i**, GTT glucose levels after 1 g kg^−1^ glucose bolus injection in male HFD-fed control and CerS2^ΔBKO^ mice at week 12 (*n* = 30 control versus 27 CerS2^ΔBKO^ mice). **j**, AUC during GTT depicted in **i**. **k**, Plasma insulin levels before injection, 20 min and 120 min after glucose injection in GTT depicted in **i** (*n* = 11 control versus 10 CerS2^ΔBKO^ mice). **l**, GTT glucose levels after 2 g kg^−1^ glucose bolus injection in male HFD-fed control and CerS2^ΔBKO^ mice at week 12 (*n* = 8 control versus 8 CerS2^ΔBKO^ mice). Dashed line indicates detection limit of glucometer. **m**, AUC of GTT depicted in **l**. **n**, Body weight of female ob/ob control and ob/ob CerS2^ΔBKO^ mice (*n* = 15 ob/ob control versus 15 ob/ob CerS2^ΔBKO^ mice). **o**, Glucose levels during GTT of female ob/ob control and ob/ob CerS2^ΔBKO^ mice at week 12 (*n* = 15 ob/ob control versus 15 ob/ob CerS2^ΔBKO^ mice). **p**, AUC of glucose levels during GTT depicted in **o**. **q**, Body weight of male ob/ob control and ob/ob CerS2^ΔBKO^ mice (*n* = 11–19 ob/ob control versus 11-19 ob/ob CerS2^ΔBKO^ mice). **r**, Glucose levels during GTT of male ob/ob control and ob/ob CerS2^ΔBKO^ mice at week 12 (*n* = 19 ob/ob control versus 17 ob/ob CerS2^ΔBKO^ mice). **s**, AUC of glucose levels during GTT depicted in **r**. Statistical analysis was performed using a two-sided Student’s *t*-test (**c**, **f**, **j**, **m**, **p** and **s**), two-way ANOVA with Sidak’s multiple comparisons test (**b**, **d**, **e**, **g**, **i**, **k**, **l**, **n**, **o** and **r**) or mixed-effects models with Sidak’s multiple comparisons test (**a**, **h** and **q**). *P* values are stated in each figure. Data points in **c**, **d**, **f**, **g**, **j**, **k**, **m**, **p** and **s** represent individual mice. Bar graphs and data points in **a**, **b**, **e**, **h**, **i**, **l**, **n**, **o**, **q** and **r** represent mean ± s.e.m. Source numerical data are available in source data. Source data

RNA sequencing indicated that CerS2 is the most abundant CerS in single human β-cells^25^ and islets of non-diabetic donors^26^ (Supplementary Fig. 1a). To directly test the hypothesis that an imbalance of VLSLs to LSLs causes β-cell dysfunction, we next aimed to decrease VLSLs specifically in pancreatic β-cells. As global CerS2 knockout mice suffer from a number of abnormalities^20,21^, we created and inter-crossed a conditional CerS2 mouse strain with Ins1-Cre mice^27^ to generate cohorts of mice lacking CerS2 specifically in β-cells (CerS2^ΔBKO^ mice). We confirmed efficient and selective ablation of CerS2 (Supplementary Fig. 1b–f). Expression of β-cell markers as well as cell stress markers were comparable in islets from control and CerS2^ΔBKO^ mice, indicating that ablation of CerS2 does not impair general β-cell development or differentiation (Supplementary Fig. 1g,h). Lipidomic analyses of CerS2^ΔBKO^ islets confirmed the expected strong decrease in VLSLs and increase in the C16:0/C24:1 ratio of Cer, HexCer and SM (Supplementary Fig. 1i–p). Hence, the CerS2^ΔBKO^ mice partially emulate the elevated C16:0/C24:1 SL ratio we observed during β-cell failure in diabetic mice (Fig. 1). Of note, VLSL levels were not decreased in plasma samples of CerS2^ΔBKO^ mice (Supplementary Fig. 1q–s), indicating that islets have a limited SL exchange with the circulation. CerS2^ΔBKO^ and control mice were phenotyped in three different paradigms of β-cell stress: normal diet feeding (ND, low insulin demand), obesogenic high-fat-diet feeding (HFD, intermediate insulin demand), and when backcrossed onto a leptin-deficient (ob/ob) background (extreme insulin demand due to massive obesity) (Fig. 2a–s and Supplementary Fig. 1t,u).

While body weight was indistinguishable (Fig. 2a), we noticed during intra-peritoneal glucose tolerance tests (GTTs) that maximum plasma glucose levels in CerS2^ΔBKO^ mice were significantly increased and returned with delay to baseline levels compared with control mice (Fig. 2b,c). Strikingly, plasma insulin levels as determined by enzyme-linked immunosorbent assay (ELISA) were reduced by 70% 20 min after glucose bolus injection (Fig. 2d), indicating impaired glucose-stimulated insulin secretion (GSIS). This phenotype was also consistently recapitulated in older CerS2^ΔBKO^ mice (Fig. 2e–g), HFD-fed CerS2^ΔBKO^ mice (Fig. 2h–m) and female and male ob/ob CerS2^ΔBKO^ mice (Fig. 2n–s and Supplementary Fig. 1u). These data unequivocally demonstrate that β-cell-specific ablation of CerS2 diminishes GSIS and systemic glucose tolerance irrespective of age, sex, body weight or diet.

**Fig. 3:**
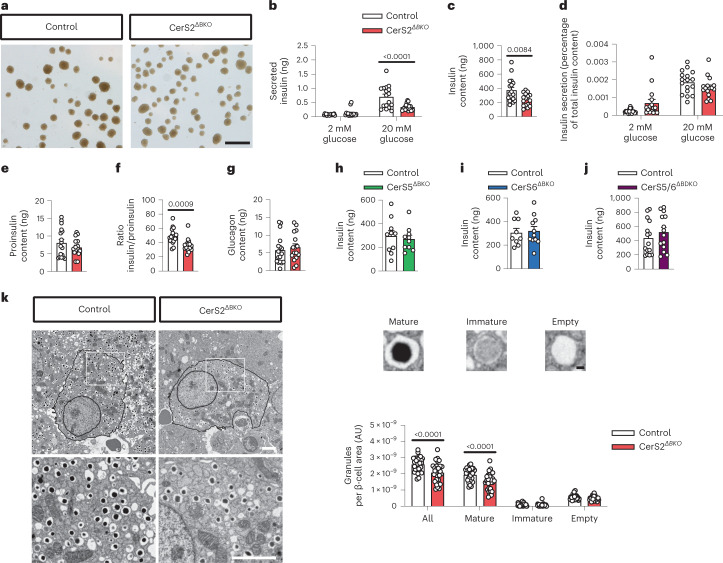
Proinsulin processing and insulin content is CerS2 dependent. **a**, Representative images of control and CerS2^ΔBKO^ islets. A lighter colour of CerS2^ΔBKO^ islets is noticeable, indicating reduced insulin content. Scale bar, 500 μm. **b**, Insulin secretion during low (2 mM) and high (20 mM) glucose static incubation of islets from male ND-fed control and CerS2^ΔBKO^ mice. Age of mice, 26 ± 2 weeks (mean ± s.d.). **c**, Insulin content of islets from male ND-fed control and CerS2^ΔBKO^ mice. **d**, Insulin secretion of islets from male ND-fed control and CerS2^ΔBKO^ mice normalized to insulin content. **e**, Proinsulin content of islets from male ND-fed control and CerS2^ΔBKO^ mice. **f**, Ratio of insulin and proinsulin content in islets from male ND-fed control and CerS2^ΔBKO^ mice. **g**, Glucagon content in islets from male ND-fed control and CerS2^ΔBKO^ mice. **h**, Insulin content of islets from male ND-fed control and CerS5^ΔBKO^ mice. Age of mice, 20 ± 2 weeks. **i**, Insulin content of islets from male ND-fed control and CerS6^ΔBKO^ mice. Age of mice, 22 ± 3 weeks. **j**, Insulin content of islets from male ND-fed control and CerS5/6^ΔBDKO^ mice. Age of mice, 18 ± 3 weeks. **k**, Representative electron microscopic pictures of isolated islets from adult male ND-fed control and CerS2^ΔBKO^ mice. Scale bars, 2 µm (β-cell) and 100 nm (magnified vesicles). Mature, immature and empty vesicles were counted and quantified by normalization to total β-cell area. *n* = islets of 17 control versus 16 CerS2^ΔBKO^ mice from four independent experiments (**b**–**g**), islets of 10 control versus 10 CerS5^ΔBKO^ mice from three independent experiments (**h**), islets of 9 control versus 11 CerS6^ΔBKO^ mice from three independent experiments (**i**) and islets of 15 control versus 14 CerS5/6^ΔBDKO^ mice from four independent experiments (**j**). For **k**, islets from four control and four CerS2^ΔBKO^ mice were pooled, respectively, and 29 individual β-cells from each pool were quantified. Statistical analysis was performed using a two-sided Student’s *t*-test (**c** and **e**–**j**) or two-way ANOVA with Sidak’s multiple comparisons test (**b**, **d** and **k**). *P* values are stated in each figure. Each data point in **b**–**j** represents the mean of three replicates of seven islets, respectively, from one individual animal. Data points in **k** represent individual β-cells. Bar graphs represent mean ± s.e.m. Source numerical data are available in source data. Source data

**Extended Data Fig. 3:**
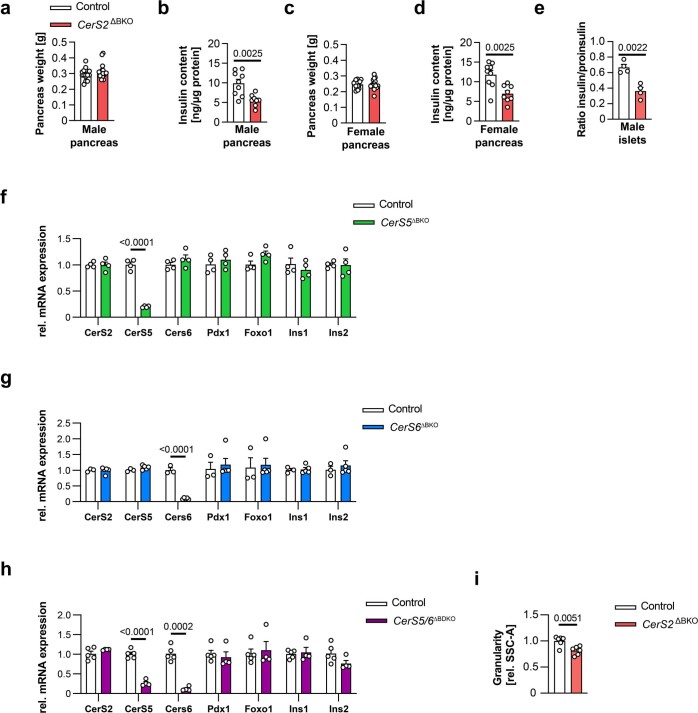
Additional phenotyping of pancreata and islets of ND-fed CerS2^ΔBKO^ mice. **a**, Pancreas weight of 16 adult male control and 16 adult male CerS2^ΔBKO^ mice. Mean age of mice, 5 months. **b**, Insulin content of pancreatic extracts of 9 adult male control and 8 adult male CerS2^ΔBKO^ mice. Mean age of mice, 8 months. **c**, Pancreas weight of 17 adult female control and 17 adult female CerS2^ΔBKO^ mice. Mean age of mice, 5 months. **d**, Insulin content of pancreatic extracts of 9 adult female control and 8 adult female CerS2^ΔBKO^ mice. Mean age of mice, 7 months. **e**, Ratio of insulin and proinsulin content in islets from 4 adult male control and 4 adult male CerS2^ΔBKO^ mice, as detected in H.L.´s laboratory after shipment of live islets from B-F.B.´s laboratory. Mean age of mice, 4 months. **f-h**, Relative mRNA expression of different CerS and beta cell identity genes in islets of control and CerS5^ΔBKO^ (f), CerS6^ΔBKO^ (g) and CerS5/6 ^ΔBKO^ (h) mice. *n* = islets of 4 control vs. 4 CerS5^ΔBKO^ mice (f); 3 control vs. 5 CerS6^ΔBKO^ mice (g); 5 control vs. 4 CerS5/6^ΔBKO^ mice (h). **i**, Beta cell granularity reflected by mean SSC-A signal of beta cells from control and CerS2^ΔBKO^ mice (*n* = 6 control vs. 6 CerS2^ΔBKO^ mice, both expressing a TdTomato reporter protein in a Cre-dependent manner). Statistical analysis was performed using unpaired two-sided Student’s *t*-test (a-e, i) or multiple two-sided *t*-tests with Holm-Sidak correction (f-h). Data points depict pancreata from unique mice (a-d), islets from individual mice (f-i) or the mean of three replicates of 15 islets, respectively, from four individual animals per genotype (in e). Bar graphs represent mean + s.e.m. *p*-values are stated in each figure. SSC-A, side scatter-area. Source numerical data are available in source data. Source data

We next considered the mechanisms leading to GSIS impairment in CerS2^ΔBKO^ mice. Indeed, one or several of the many molecular pathways involved in insulin synthesis, maturation and secretion could theoretically be impaired in CerS2^ΔBKO^ β-cells. Islets from ND-fed CerS2^ΔBKO^ mice consistently appeared lighter in colour, suggesting reduced insulin content (Fig. 3a). While basal insulin secretion was comparable, GSIS was reduced by approximately 40% in CerS2^ΔBKO^ islets from ND-fed mice in static incubation experiments (Fig. 3b). Importantly, we detected a significant reduction in islet as well as pancreatic insulin content (Fig. 3c and Extended Data Fig. 3a–d). When normalized to insulin content, relative insulin secretion from stimulated CerS2^ΔBKO^ islets was no longer different from control islets (Fig. 3d), indicating that reduced CerS2^ΔBKO^ β-cell insulin content, rather than primarily a secretion defect, is the key trigger of this phenotype. Importantly, whereas proinsulin levels were unaltered, the ratio of insulin to proinsulin was significantly reduced (Fig. 3e,f and Extended Data Fig. 3e), arguing for reduced processing of proinsulin to insulin in CerS2^ΔBKO^ islets as a potential underlying mechanism. In line with the selective ablation of CerS2 in β-cells, glucagon content was unchanged in CerS2^ΔBKO^ islets (Fig. 3g). To assess if ablation of LSLs in β-cells would also affect insulin content, we generated and phenotyped mice lacking CerS5 (ref. ^6^), or CerS6 (ref. ^5^), or both CerS5 and CerS6 in β-cells (Extended Data Fig. 3f–h). Insulin content in islets of ND-fed adult animals of each of these strains was unaltered (Fig. 3h–j). Next, we imaged islets by transmission electron microscopy and observed a quantitative reduction of vesicles containing mature insulin in CerS2^ΔBKO^ islets (Fig. 3k), which was mirrored by quantification of β-cell granularity by flow cytometry (Extended Data Fig. 3i). These data demonstrate that ablation of the VLSL-synthesizing enzyme CerS2 causes a reduction in the insulin-to-proinsulin ratio.

**Fig. 4:**
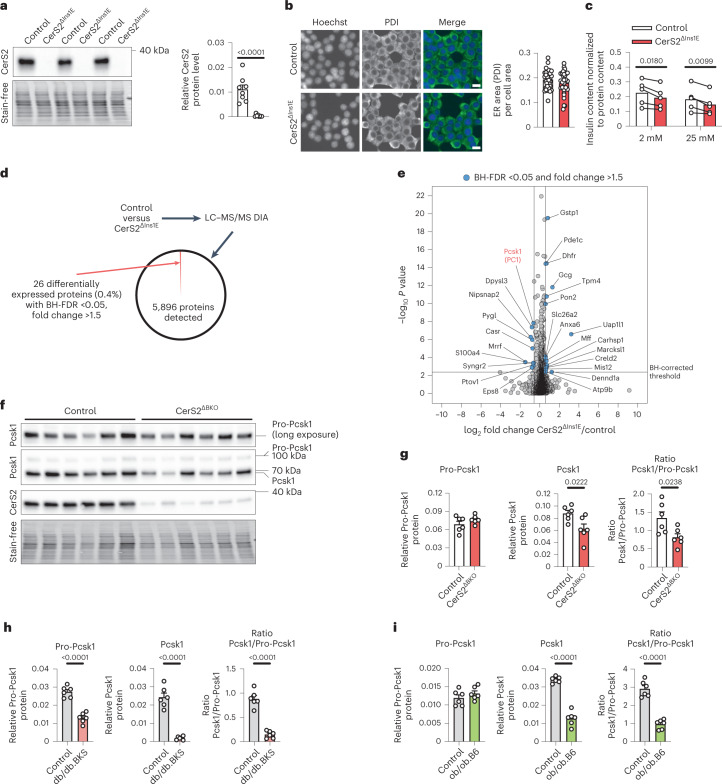
Abundance of the proinsulin processing enzyme Pcsk1 is CerS2 dependent. **a**, Verification of loss of CerS2 in CerS2^ΔIns1E^ cells by immunoblot. Left: representative immunoblot. Right: quantification of Cers2 signals (*n* = 8 independent experiments). **b**, Representative immunostaining (left) and quantification (right) of ER marker PDI in control and CerS2^ΔIns1E^ cells (*n* = 36 control versus 28 CerS2^ΔIns1E^ well sites from one experiment). Scale bar, 10 μm. **c**, Quantification of insulin content in control and CerS2^ΔIns1E^ cells at low (2 mM) and high (25 mM) glucose levels (*n* = 5 independent experiments). **d**, Experimental design and results for proteome analyses in control and CerS2^ΔIns1E^ cells. **e**, Volcano plot showing log_2_ fold change of proteins between CerS2^ΔIns1E^ and control cells plotted against the −log_10_ *P* value of a two-sided paired Student’s *t*-test. BH-FDR <0.05 and fold change >1.5 was used as significance cut-offs (*n* = 3 control versus 3 CerS2^ΔIns1E^ samples collected in three independent experiments). **f**,**g**, Immunoblot detection of Pro-Pcsk1 and Pcsk1 protein levels in islets of control and CerS2^ΔBKO^ mice. **f**, Representative immunoblot. Each lane represents islets of one individual mouse. **g**, Quantification of Pro-Pcsk1 (left) and Pcsk1 (middle) protein levels and ratio of Pcsk1/Pro-Pcsk1 (right; *n* = islets of six control versus six CerS2^ΔBKO^ mice). **h**, Immunoblot detection of Pro-Pcsk1 and Pcsk1 protein levels in islets of 12-week-old control and db/db.BKS mice. Quantification of Pro-Pcsk1 (left) and Pcsk1 (middle) protein levels and ratio of Pcsk1/Pro-Pcsk1 (right; *n* = islets of six control versus six db/db.BKS mice). Representative immunoblot is shown in Extended Data Fig. 4d. **i**, Immunoblot detection of Pro-Pcsk1 and Pcsk1 protein levels in islets of 12-week-old control and ob/ob.B6 mice. Quantification of Pro-Pcsk1 (left) and Pcsk1 (middle) protein levels and ratio of Pcsk1/Pro-Pcsk1 (right; *n* = islets of six control versus six ob/ob.B6 mice). Representative immunoblot is shown in Extended Data Fig. 4e. Statistical analysis was performed using a two-sided Student’s *t*-test (**a**, **b**, **g**, **h** and **i**) and two-way ANOVA with Sidak’s multiple comparisons test (**c**). *P* values are stated in each figure. Bar graphs represent mean (**c**) or mean ± s.e.m. (**a**, **b**, **g**, **h** and **i**). Connecting lines indicate both samples are from one experiment. Data points in **b** represent individual well sites. Data points in **a** and **c** represent independent experiments. Data points in **g**–**i** represent islets from individual mice. Stain-free signal was used for normalization of all immunoblots. Source numerical data and unprocessed blots are available in source data. Source data

**Extended Data Fig. 4:**
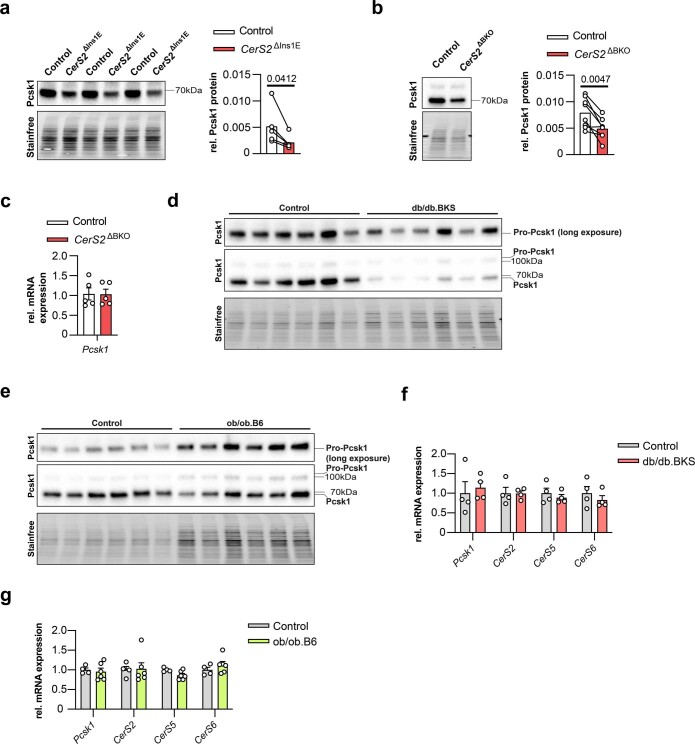
Pro-Pcsk1 and Pcsk1 protein abundance in CerS2-deficient cells and islets of metabolically impaired mouse models. **a**, Quantification of Pcsk1 levels in control and CerS2^ΔIns1E^ cells by CrispR-verified antibody (Supplementary Fig. 2o, Cell Signaling #11914, discontinued). Left, representative immunoblot. Right, quantification of Pcsk1 signals (*n* = 5 independent experiments). **b**, Quantification of Pcsk1 protein levels in control and CerS2^BKO^ islets by CrispR-verified antibody (Supplementary Fig. 2o, Cell Signaling #11914, discontinued). Left, representative immunoblot. Right, quantification of Pcsk1 signals (*n* = 8 independent experiments). **c**, Quantification of Pcsk1 mRNA levels in islets from male control and CerS2^BKO^ mice by qPCR (*n* = 5 independent experiments). **d**, Representative immunoblots to Fig. 4h. **e**, Representative immunoblots to Fig. 4i. **f, g**, Quantification of mRNA levels of Pcsk1 and various CerS in islets of 12 week old control and db/db.BKS mice (f, *n* = 4 control and 4 db.db/BKS islet samples) and islets of 12 week old control and ob/ob.B6 mice (g, *n* = 4 control and 6 ob/ob.B6 islets samples). Statistical analysis was performed using a paired two-sided Student’s *t*-test (a, b), two-sided Student’s *t*-test (c) and two-sided multiple *t*-tests with Holm-Sidak correction (f, g). *P*-values are stated in each figure. Bar graphs represent means (a, b) or means + s.e.m. (c, f, g). Connecting lines indicate both samples are from one experiment. Data points represent independent experiments (a), islets from individual mice (c) or individual islet samples (f, g). Stain-Free signal was used for normalization of all immunoblots. Source numerical data and unprocessed blots are available in source data. Source data

**Extended Data Fig. 5:**
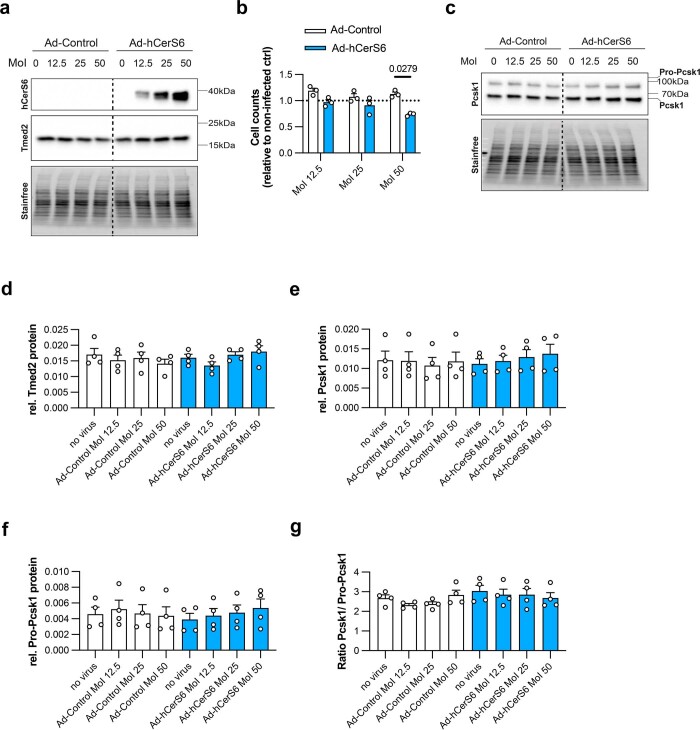
Adenoviral CerS6 overexpression does not affect Pcsk1 levels. **a**, Immunoblot detection of human CerS6 (hCerS6) and Tmed2 protein levels in INS1E cells after overexpression of hCerS6 via adenovirus for 48 h (representative immunoblot). **b**, Ins1E cell counts after 48 hours of infection with a control adenovirus or hCerS6-expressing adenovirus (*n* = 3 independent experiments). A reduction of cell counts is in line with the ability of CerS6 overexpression to induce apoptosis in several cell types. **c**, Immunoblot detection of Pro-Pcsk1 and Pcsk1 protein levels (Cell Signaling #18030) in Ins1E cells infected with a control adenovirus or hCerS6-expressing adenovirus for 48 h (representative immunoblot). **d-g**, Quantification of Tmed2 (d), Pcsk1 (e), Pro-Pcsk1 signals (f) and Pcsk1/ Pro-Pcsk1 ratio (g) from 4 independent experiments. Statistical analysis was performed using two-way ANOVA with Sidak’s multiple comparisons test (b, d-g). Data points in (b, d-g) represent independent experiments. Bar graphs in (b, d-g) represent means + s.e.m. Stain-Free signal was used for normalization of all immunoblots. Source numerical data and unprocessed blots are available in source data. Source data

We reasoned that CerS2 ablation could affect one or more key proteins involved in the complex insulin processing machinery^1^. To identify relevant proteins in an in vitro model system with total loss of CerS2, we employed clustered regularly interspaced short palindromic repeats (CRISPR)/Cas9 to knock out CerS2 in the rat pancreatic β-cell line Ins1E, which as expected reduced VLSL levels (CerS2^ΔIns1E^ cells; Fig. 4a and Supplementary Fig. 2a–i)^28,29^. In contrast to other cell types with reduced CerS2 abundance^30,31^, CerS2^ΔIns1E^ cells showed no marked signs of altered basic cellular functions (Fig. 4b and Supplementary Fig. 2j–n) and, similar to our finding in CerS2^ΔBKO^ islets, demonstrated a reduction in insulin content (Fig. 4c). We performed mass spectrometry (MS)-based protein quantification of control and CerS2^ΔIns1E^ cell samples. CerS2 ablation caused a highly selective change in cellular processes, as only 0.4% of proteins were ≥1.5-fold up- or downregulated (Fig. 4d,e and Supplementary Table 7). Among several interesting candidates, we noticed that Pcsk1 (a crucial enzyme in proinsulin-to-insulin conversion) was decreased by approximately 50% in CerS2^ΔIns1E^ cells (Fig. 4e). We pursued this observation, since loss of just one Pcsk1 allele has been previously shown to impair proinsulin processing^9^. Quantification of immunoblots using a validated antibody (Supplementary Fig. 2o) confirmed a reduction in Pcsk1 expression levels in both CerS2^ΔIns1E^ cells and CerS2^ΔBKO^ islets, providing evidence that CerS2 influences Pcsk1 protein expression in independent experimental systems (Extended Data Fig. 4a,b). Of note, Pcsk1 mRNA levels in CerS2^ΔBKO^ islets were unaffected (Extended Data Fig. 4c). Pcsk1 is synthesized as an immature Pro-Pcsk1 protein. During its translocation from endoplasmic reticulum (ER) to Golgi to secretory vesicles a complex series of post-translational modifications are thought to stimulate cleavage at both N- and C-termini, resulting in the enzymatically active (mature) Pcsk1 protein found in insulin-secretory vesicles^32^. With the help of a second antibody able to detect both Pro-Pcsk1 and mature Pcsk1 (Supplementary Fig. 2p), we confirmed that, in CerS2^ΔBKO^ islets, levels of mature Pcsk1 protein were reduced, whereas protein levels of Pro-Pcsk1 were not, as also indicated by the Pcsk1/Pro-Pcsk1 ratio (Fig. 4f,g). In contrast, adenoviral overexpression of CerS6 had no effect on Pcsk1 abundance or the Pcsk1/Pro-Pcsk1 ratio in Ins1E cells (Extended Data Fig. 5a–g). In islets of db/db.BKS mice, levels of both protein forms as well as the Pcsk1/Pro-Pcsk1 ratio were reduced, a phenotype that was partially ameliorated in ob/ob.B6 islets (Fig. 4h,i and Extended Data Fig. 4d–g). These data indicate that a selective reduction in mature Pcsk1 levels is involved in the observed dysregulation of proinsulin processing in CerS2-deficient β-cells.

**Fig. 5:**
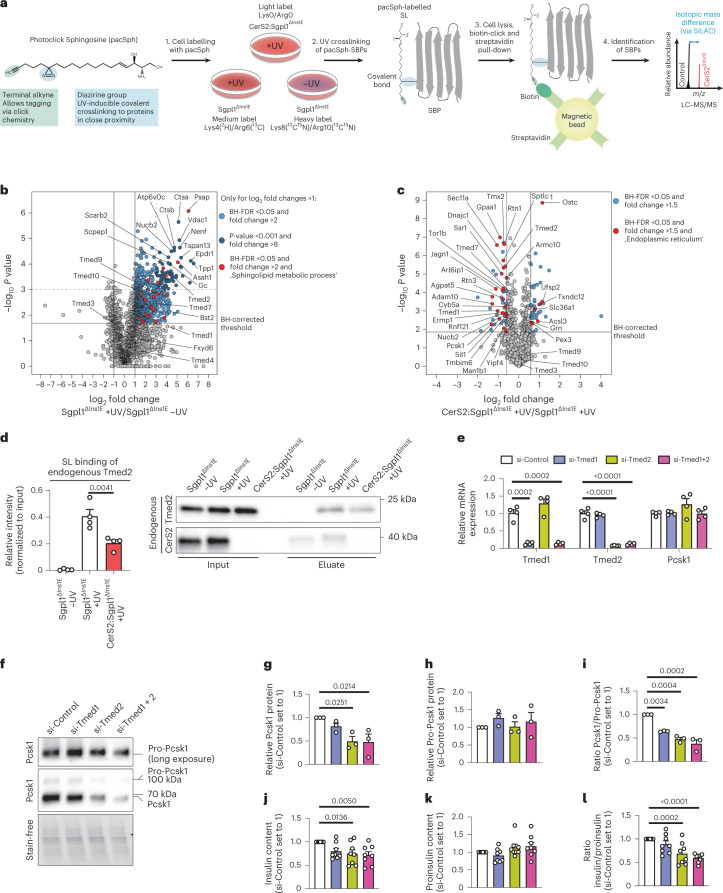
SL–protein interactomics reveals a role for Tmed2 in proinsulin processing. **a**, Experimental setup for identification of SBPs in a SILAC-based approach. pacSph treatment of Sgpl1^ΔIns1E^ and CerS2:Sgpl1^ΔIns1E^ cells differentially labelled with stable isotopes allows crosslinking of SL-protein complexes by UV irradiation (with omission of UV irradiation as a control condition), followed by cell lysis and conjugation of biotin to the SL-protein complexes. After Streptavidin-based pull-down, SBPs can be identified and quantified in the same MS run by the differing peptide mass due to SILAC isotope labelling. **b**, Volcano plot showing log_2_ fold change of proteins pulled down from pacSph-treated Sgpl1^ΔIns1E^ (+UV) versus Sgpl1^ΔIns1E^ (−UV) cells plotted against the −log_10_ *P* values of a one-sample two-sided *t*-test against 0. Proteins with log_2_ fold change >1 and a BH-FDR <0.05 are regarded as SBPs (*n* = 4 independent experiments). **c**, Volcano plot showing log_2_ fold change of SBPs identified in **b** (Supplementary Fig. 3e) and pulled down from pacSph-treated CerS2:Sgpl1^ΔIns1E^ (+UV) versus Sgpl1^ΔIns1E^ (+UV) cells plotted against the −log_10_ *P* values of a two-sample two-sided equal variance *t*-test (*n* = 4 independent experiments). SBPs with a fold change >1.5 and a BH-FDR <0.05 were regarded as Cers2-dependent SBPs. Fold enrichment was 6.55 and FDR-corrected *P* value was 3.12^−10^ for GO term ‘endoplasmic reticulum’. **d**, pacSph pull-down of endogenous Tmed2 in Sgpl^ΔIns1E^ and Sgpl1:CerS2^ΔIns1E^ cells (*n* = 4 independent experiments); exemplary immunoblot (right) and quantification (left). Eluate intensities were normalized to respective input intensities. **e**, Relative mRNA expression of Tmed1, Tmed2 and Pcsk1 in murine pseudoislets transfected with control siRNA or siRNA against Tmed1, Tmed2 or both. *n* = 4 independent experiments. **f**–**i**, Immunoblot detection of Pro-Pcsk1 and Pcsk1 protein levels in pseudoislets transfected with siRNA as described in Extended Data Fig. 8e; *n* = 3 independent experiments. **f**, Representative immunoblot. **g**, Quantification of Pcsk1. **h**, Quantification of Pro-Pcsk1. **i**, Ratio of Pcsk1 to Pro-Pcsk1. **j**–**l**, Insulin content (**j**), proinsulin content (**k**) and ratio of insulin to proinsulin (**l**) in pseudoislets transfected with siRNA as described in Extended Data Fig. 8e determined via ELISA (*n* = 8 independent experiments). Statistical analyses were performed using one-way ANOVA with Tukey’s multiple comparisons test (**d**) and repeated measures one-way ANOVA with Tukey’s multiple comparisons test (**e** and **g**–**l**). In **e**, ANOVA was performed for each mRNA target individually. *P* values are stated in each figure. Data points in **d**, **e** and **g**–**l** represent individual experiments. Bar graphs represent mean ± s.e.m. For one experiment in **j**–**l**, the mean of five replicates, consisting of nine pseudoislets, respectively, was plotted per condition. Stain-free signal was used for normalization of immunoblots in **g**–**i**. Source numerical data and unprocessed blots are available in source data. Source data

**Extended Data Fig. 6:**
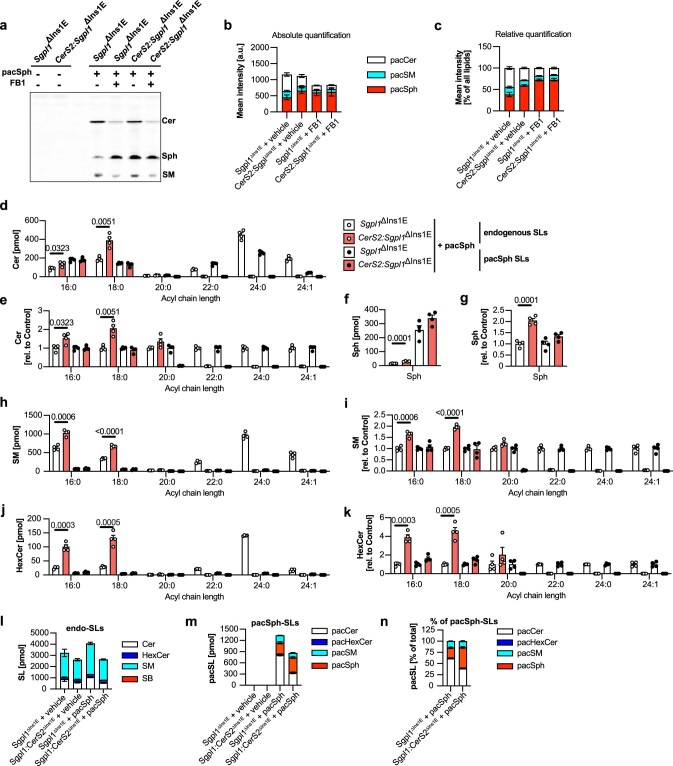
pacSph incorporation into sphingolipids during PACS interactomics. **a-c**, Thin layer chromatography (TLC) analyses of pacSph incorporation in Sgpl1^ΔIns1E^ and CerS2:Sgpl1^ΔIns1E^ cells. **a**, Exemplary TLC image. **b**, Quantification of pacSphingosine (pacSph), pacSph-derived Ceramides (pacCer) and pacSph-derived sphingomyelins (pacSM) in Sgpl^ΔIns1E^ and CerS2:Sgpl1^ΔIns1E^ cells treated with Fumonisin B1 (FB1) or vehicle, respectively, and 5 µM pacSph for 1 h (*n* = 6 independent experiments). **c**, Relative quantification of all pacSLs shown in (b). The sum of all pacSLs per sample was set to 100%. **d-n**, MS-based quantification of pacSph incorporation in Sgpl1^ΔIns1E^ and CerS2:Sgpl1^ΔIns1E^ cells. **d, e**, Absolute (d) and relative (e) quantification of endogenous (white dots) and pacSph-derived ceramides (black dots) in cells treated with 5 µM pacSph for 1 h (*n* = 4 independent experiments). **f, g**, Absolute (f) and relative (g) quantification of endogenous sphingosine (Sph) and pacSph as described for (d) and (e). **h, i**, Absolute (h) and relative (i) quantification of endogenous and pacSph-derived sphingomyelins as described for (d) and (e). **j, k**, Absolute (j) and relative (k) quantification of endogenous and pacSph-derived hexosylceramides as described for (d) and (e). **l**, Sum of all measured endogenous sphingoid bases (SB = sphingosine and sphinganine), ceramides (Cer), sphingomyelins (SM) and hexosylceramides (HexCer). **m**, Sum of all measured pacSph-derived ceramides, sphingomyelins, hexosylceramides (pacHexCer) and pacSph in cells described in (l). **n**, Relative amounts of pacSph-derived SLs in pacSph-treated cells described in (l) and (m). The sum of all pacSLs per sample was set to 100%. Statistical analysis in (d-k) was performed separately for endogenous SLs and pacSph SLs using two-sided multiple *t*-tests with Holm-Sidak correction. For VLSLs, no statistical test was performed due to ablation of CerS2. Data points represent independent experiments. Bar graphs represent means + s.e.m. Pac-, photoactivatable and clickable-; MS, mass spectrometry. Source numerical data and unprocessed blots are available in source data. Source data

**Extended Data Fig. 7:**
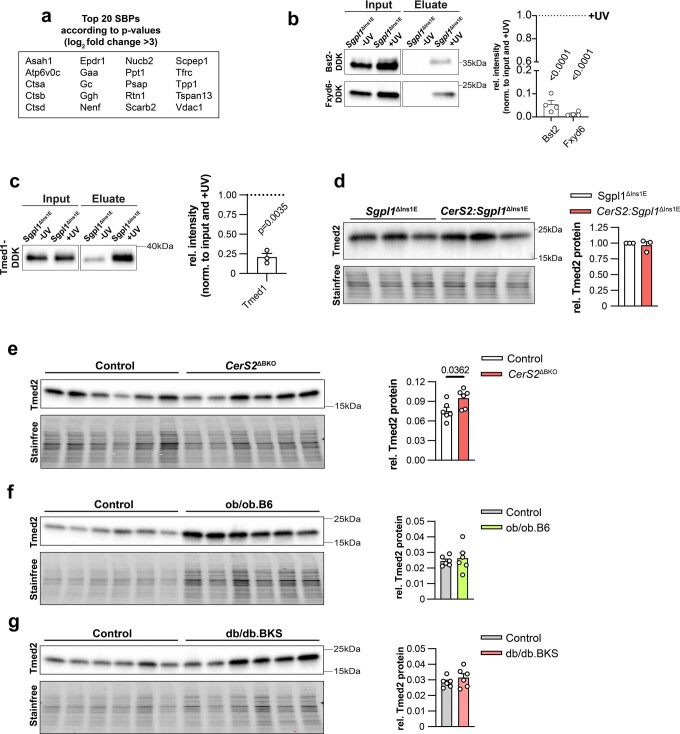
SBP confirmation and Tmed2 abundance. **a**, Top 20 SBPs identified in Fig. 5b according to *p*-values and a log_2_ fold change > 3. **b**, Verification of Bst2 and Fxyd6 as SBPs by overexpression of DDK-tagged variants in Sgpl1^ΔIns1E^ cells followed by pacSph-pulldown; representative immunoblots (left) and quantification (right). Eluate band intensities were normalized to input bands and +UV samples were set to 1 (*n* = 4 independent experiments). **c**, Verification of Tmed1 as SBP as described in (b); representative immunoblot (left) and quantification (right). *n* = 3 independent experiments. **d**, Immunoblot detection of Tmed2 protein levels in Sgpl1^ΔIns1E^ and Cers2:Sgpl1^ΔIns1E^ cells. Representative immunoblot showing 3 replicates per genotype (left) and quantification (right). *n* = 3 independent experiments with 3 replicates per genotype, respectively. **e**, Immunoblot detection of Tmed2 protein levels in islets of 6 control and 6 Cers2^ΔBKO^ mice. Representative immunoblot (left) and quantification (right). **f**, Immunoblot detection of Tmed2 protein levels in islets of 6 control and 6 ob/ob.B6 mice at week 12. Representative immunoblot (left) and quantification (right). **g**, Immunoblot detection of Tmed2 protein levels in islets of 6 control and 6 db/db.BKS mice at week 12. Representative immunoblot (left) and quantification (right). Statistical analysis was performed using a one sample *t*-test against 1 (b, c) or Student’s *t*-test (d-g). *P*-values are stated in each figure. Bar graphs represent means + s.e.m. Data points in (b, c and d) represent individual experiments. Data points in (e-g) represent islets from individual mice. Stain-Free signal was used for normalization of all immunoblots, except for (b) and (c). Stain-Free images of (e), (f) and (g) were reproduced from Fig. 4f, Extended Data Fig. 4e, d, as the same PVDF membranes were used for detection of Pro-Pcsk1, Pcsk1 and Tmed2, respectively. Source numerical data and unprocessed blots are available in source data. Source data

**Extended Data Fig. 8:**
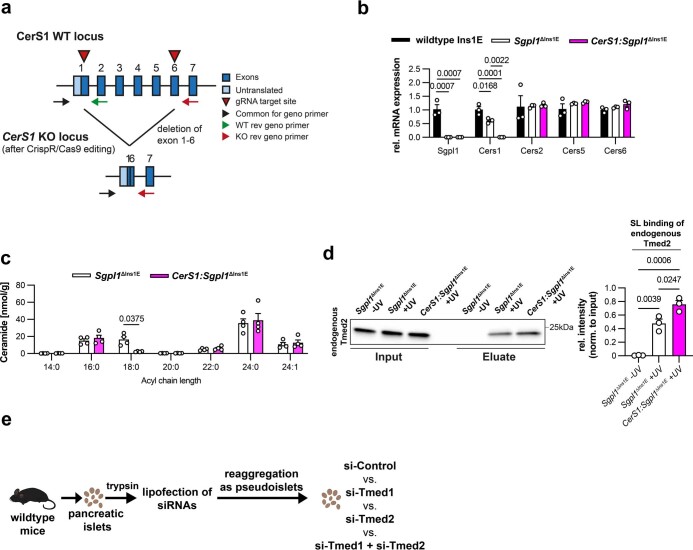
Tmed2:SL interaction upon CerS1 deficiency. **a**, Double cut CrispR/Cas9 knockout strategy for CerS1 in Ins1E cells. **b**, Relative mRNA expression of Sgpl1 and various CerS in wildtype Ins1E cells, Sgpl1^ΔIns1E^ and CerS1:Sgpl1^ΔIns1E^ cells. *n* = 3 independent experiments. Wildtype Ins1E cDNA was also used for qPCR in Supplementary Fig. 3c. **c**, Quantification of ceramides of various acyl chain lengths in Sgpl1^ΔIns1E^ and CerS1:Sgpl1^ΔIns1E^ cells by targeted lipidomics (*n* = 4 independent experiments). Lipid amounts were normalized to cell pellet weights. **d**, pacSph pull-down of endogenous Tmed2 in Sgpl1^ΔIns1E^ and CerS1:Sgpl1^ΔIns1E^ cells (*n* = 3 independent experiments); exemplary immunoblot (left) and quantification (right). Eluate intensities were normalized to respective input intensities. **e**, Experimental setup for knock-down of Tmed1, Tmed2 and both in murine pseudoislets. Statistical analysis was performed using one-way ANOVA with Tukey’s multiple comparisons test for each individual mRNA target in (b), multiple two-sided *t*-tests with Holm-Sidak correction in (c) and repeated measures one-way ANOVA with Tukey’s multiple comparisons test in (d). Data points represent independent experiments. Bar graphs represent means + s.e.m. The Sgpl1^ΔIns1E^ and CerS1:Sgpl1^ΔIns1E^ cells are pools of 3 individual monoclonal cell lines, respectively. Source numerical data and unprocessed blots are available in source data. Source data

To examine how CerS2 selectively affects mature Pcsk1 protein abundance, we next tested the hypothesis that altered interactions of sphingolipid-binding proteins (SBPs) with VLSLs may cause the reduction in Pcsk1 and insulin content in CerS2-deficient β-cells. This hypothesis was in part based on the suggestion that Pcsk1 interacts with membrane lipids^33^, which may increase its chance to interact with SBPs. To first define the β-cell SL–protein interactome, we performed a chemoproteomic screen in which a chemically modified non-toxic (Supplementary Fig. 3a) SL precursor (photoactivatable and clickable sphingosine, pacSph) was fed to cells for 1 h to be rapidly incorporated into de novo synthesized SLs (Extended Data Fig. 6a–n and Supplementary Table 8). Ultraviolet (UV) irradiation covalently crosslinked cellular SL–protein complexes, allowing for subsequent MS-based identification and quantification of SL-interacting proteins. Knockout of the SL-specific catabolic enzyme sphingosine-1-phosphate-lyase 1 (Sgpl1) ensured that only SLs were labelled with pacSph^34^ (Supplementary Fig. 3b–d). Furthermore, stable isotope labelling^35^ allowed direct comparison between SBPs in Sgpl1^ΔIns1E^ and CerS2:Sgpl1^ΔIns1E^ cells in the same MS run (for workflow, see Fig. 5a). This study revealed 1,045 proteins, including several SL metabolic enzymes to be significantly enriched (at least two-fold, Benjamini–Hochberg false discovery rate (FDR) <0.05) in +UV versus −UV treated Sgpl1^ΔIns1E^ cells and/or +UV CerS2:Sgpl1^ΔIns1E^ cells versus −UV treated Sgpl1^ΔIns1E^ cells, and six additional proteins were detected only in +UV samples, not in −UV samples (Fig. 5b,c, Extended Data Fig. 7a, Supplementary Fig. 3e and Supplementary Tables 9 and 10). We validated our dataset by overexpression of DDK-tagged proteins and subsequent UV-dependent pull-down in Sgpl1^ΔIns1E^ cells (Extended Data Fig. 7b). Next, we assessed the VLSL-dependent SL–protein interactome (CerS2:Sgpl1^ΔIns1E^ versus Sgpl1^ΔIns1E^ cells, both UV treated). While the vast majority (approximately 91%) of all detected proteins continued to interact with SLs, only 50 proteins significantly decreased and 43 proteins increased their interactions with SLs following ablation of CerS2 (fold change >1.5, BH-FDR <0.05; Fig. 5c and Supplementary Tables 11 and 12). Notably, Gene Ontology (GO)-term analysis revealed that proteins associated with the term ‘endoplasmic reticulum’ were overrepresented in the list of proteins with reduced SL interaction in CerS2:Sgpl1^ΔIns1E^ cells. Further analysis of the interactome data revealed that six members of the transmembrane emp24 domain-containing protein (Tmed) family were identified as SBPs (Fig. 5b and Supplementary Fig. 3e), and two of them, Tmed1 and Tmed2, interacted with SLs in a CerS2-dependent manner (Fig. 5c). We next focused on Tmed1 and Tmed2 as CerS2-dependent SBPs, since Tmed2 is involved in both COPII- and COPI-mediated shuttling of ER and Golgi proteins^36^, and can interact with several lipid species, including with C18:0 SM, a CerS1-dependent lipid^37,38^. Tmed1 was confirmed as an SBP in overexpression experiments (Extended Data Fig. 7c). Remarkably, we directly demonstrated that approximately 50% of the interaction of endogenous Tmed2 with SLs is CerS2 dependent in living β-cells (Fig. 5d). This reduced interaction was not due to any change in Tmed2 protein level (Extended Data Fig. 7d–g). Surprisingly, ablation of CerS1 increased SL interaction of Tmed2, which may indicate that SL–SBP interactions are cell-type specific (Extended Data Fig. 8a–d).

**Extended Data Fig. 9:**
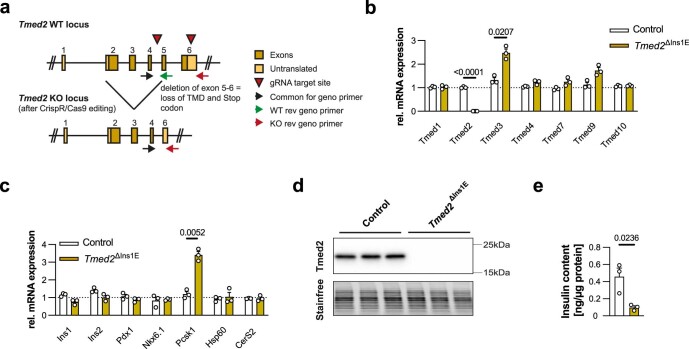
Reduced insulin levels in Tmed2-deficient Ins1E cells. **a**, Double cut CrispR/Cas9 knockout strategy for Tmed2 in Ins1E cells. **b, c**, Relative mRNA expression of various Tmed family members (b) and beta cell identity markers (c) in wildtype Ins1E, control and Tmed2^ΔIns1E^ cells. Wildtype Ins1E samples were set to 1 (dotted line). *n* = 3 independent experiments. Note that potentially as a sign of attempted compensation, Pcsk1 mRNA levels are increased in Tmed2-deficient Ins1E cells. **d**, Representative immunoblot analysis of Tmed2 protein expression in 3 control vs. 3 Tmed2^ΔIns1E^ replicate lysates. **e**, Insulin content in control and Tmed2^ΔIns1E^ cells determined via ELISA. *n* = 3 independent experiments with 3 replicates per genotype, respectively. Statistical analysis was performed using multiple two-sided *t*-tests with Holm-Sidak correction (b and c) and a two-sided Student’s *t*-test (e). Data points represent independent experiments. Bar graphs represent means + s.e.m. The control and Tmed2^ΔIns1E^ cells are pools of individual monoclonal cell lines, respectively (8 monoclonal control cell lines and 3 monoclonal Tmed2^ΔIns1E^ cell lines were used for pooling). Source numerical data and unprocessed blots are available in source data. Source data

**Extended Data Fig. 10:**
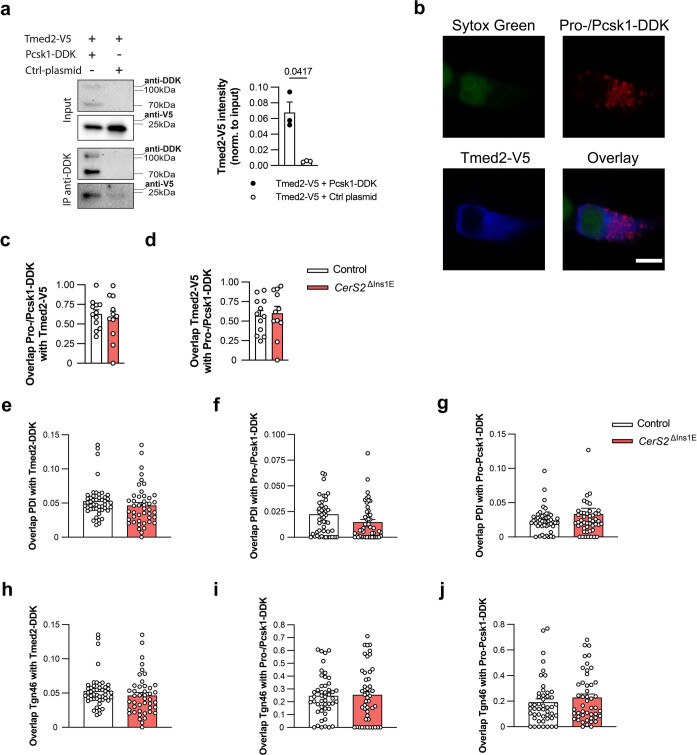
Interaction and localization of overexpressed Tmed2 and Pcsk1 in Ins1E cells. **a**, Co-immunoprecipitation (Co-IP) of co-overexpressed Tmed2-V5 and Pro-Pcsk1/Pcsk1-DDK in Ins1E cells. Representative immunoblot (left) and quantification of three replicate experiments (right). As Ctrl-plasmid, the promotorless pNL1.3 from Promega (N1021) was used. **b**, Representative confocal images for co-localization of overexpressed Tmed2-V5 and Pro-/Pcsk1-DDK in Ins1E cells. Green, SytoxGreen as nucleus marker; red, Pro-/Pcsk1-DDK; blue, Tmed2-V5. Scale bar, 5 µm. **c, d**, Quantification of overlap of Pro-/Pcsk1-DDK with Tmed2-V5 (c) and Tmed2-V5 with Pro-/Pcsk1-DDK (d) in control and CerS2^ΔIns1E^ cells. *n* = 2 independent experiments; only one experiment shown. **e-j**, Overlap of ER-marker PDI and Golgi-Marker TGN46 with Tmed2-V5, Pro-/Pcsk1-DDK (allowing detection of both Pro-Pcsk1 as well as mature Pcsk1) and Pro-Pcsk1 (only allowing detection of the immature Pro-Pcsk1 protein) after overexpression in control and CerS2^ΔIns1E^ cells. *n* = 3 independent experiments. Statistical analysis was performed using a paired two-sided Student’s *t*-test (a) and unpaired two-sided Students *t*-tests (c-j). Data points represent replicate experiments (a) and individually quantified cells (c-d) or well sites (e-j). Bar graphs represent means + s.e.m. Source numerical data and unprocessed blots are available in source data. Source data

To directly investigate if Tmed1 and/or Tmed2 functionally affect insulin processing, we transfected small interfering RNA (siRNAs) against Tmed1, Tmed2 or both into trypsinized wild-type islet cells, followed by gravity-assisted re-aggregation into pseudoislets^39^ (Fig. 5e, Extended Data Fig. 8e and Supplementary Fig. 3f). Tmed2 and combined Tmed1 + Tmed2, but not Tmed1 knockdown alone, was sufficient to selectively reduce the protein level of mature Pcsk1 as well as the Pcsk1/ProPcsk1 ratio (Fig. 5f–i), and resulted in reduced insulin levels and a lower insulin/proinsulin ratio (Fig. 5j–l). In line with this, knockout of Tmed2 reduced insulin levels in Ins1E cells (Extended Data Fig. 9a–e). We hypothesize that VLSL binding of Tmed2 promotes (Pro-)Pcsk1 transport towards insulin-secretory vesicles, as both proteins are able to interact and co-localize, as determined by co-overexpression experiments (Extended Data Fig. 10). Interestingly, among several types of β-cell stress, only acute induction of dysfunctional ER–Golgi protein transport in human islets decreased CerS2 expression (Supplementary Fig. 3g–j). Moreover, aberrant Golgi protein trafficking is a recently discovered hallmark of some forms of monogenic diabetes^40^, type 1 diabetes and T2D^41^. In sum, our findings suggest that VLSLs and Tmed2 alter mature Pcsk1 protein levels, and this may provide a partial explanation for the proinsulin processing defect observed in T2D (Supplementary Fig. 3k). In addition to targeting SL synthesis with small molecules^42,43^, modifying SL-SBP interactions with orally available synthetic SL analogues^44^ could be an alternative therapeutic approach to improve β-cell function in diabetes.

## Methods

### Mouse models and knockout mouse strains

All animal procedures were approved by the Department for Environment and Consumer Protection of North Rhine-Westphalia, Germany (LANUV; #84.02.04.2017.A305 and #81-02.04.2019.A321) and the DDZ Institutional Animal Welfare Committee. Mice were killed in accordance with the German Animal Protection Law (TSchG). The following mouse strains (including controls) were commercially acquired: ob/ob.B6 (Jackson Laboratories, USA/JAX #000632), db/db.BKS (JAX #000642), Akita mice (JAX #003548) and C57BL/6JRj (Janvier). For generation of CerS2^ΔBKO^, CerS5^ΔBKO^, CerS6^ΔBKO^ and CerS5/6^ΔBDKO^ mouse cohorts, mice carrying the Ins1-Cre Knockin^27^ (on a C57BL/6J background) were crossed with the respective conditional allele(s)^5,6^. The CerS2 conditional allele was generated (loxP sites flanking exons 2–11) as reported for the CerS5 and CerS6 alleles^5,6^. For reporter studies, a conditional red fluorescent reporter strain^45^ (JAX #007909) was crossed into the CerS2^ΔBKO^ strain. Mice carrying one Cre allele were used as controls in all experiments except the ob/ob cohorts, in which controls were Cre-negative ob/ob CerS2^fl/fl^ littermates. Male adult mice were used for experiments except when stated otherwise. The animals were maintained on a 12 h light/dark cycle (lights on from 6:00 to 18:00) and had ad libitum access to tap water and a standard rodent chow (58% carbohydrates, 33% protein, 9% fat, R/M−H Extrudat, ssniff Spezialdiäten GmbH, Soest, Germany) or high-fat diet (60% fat; #D12492, Research Diets).

### Insulin and glucose tolerance tests

Insulin tolerance tests and GTTs were performed following standard protocols^46,47^. Mice that did not reach 100 mg dl^−1^ blood glucose levels 20 min after glucose injection were excluded from analysis (two ND-fed male CerS2^ΔBKO^ mice in GTT week 12; one ND-fed male control and one CerS2^ΔBKO^ mouse in GTT week 16). Plasma insulin was quantified using the ultrasensitive rat insulin ELISA (#90062, Crystal Chem).

### Islet preparations

Islets were collected according to standard protocols^47^, with the following modifications: for some isolations, islets were centrifuged in layers of 3 ml Histopaque 1119 (bottom layer, #11191, Sigma-Aldrich), 3 ml Histopaque 1083 (#10831, Sigma-Aldrich), 3 ml Histopaque 1077 (#10771, Sigma-Aldrich, or Lymphoprep (#07801, Stemcell Technologies)) and 3 ml RPMI (Thermo Fisher, #11835030 or #32404014) without serum (top layer). Islets were kept in full islet medium (DMEM (Life Technologies #11880036) containing 11 mM d-glucose, 2 mM Na-pyruvate, 2 mM Glutamax, 0.18 mM 2-mercaptoethanol, 11 mM HEPES, 10% fetal calf serum (FCS) and 100 U ml^−1^ penicillin–streptomycin (all Gibco by Life Technologies)). Islets were imaged using a Nikon SMZ1270 stereomicroscope using a Nikon DFK 23U274 camera (camera device driver from The Imaging Source Europe GmbH).

### RNA interference in primary mouse islets and generation of pseudoislets

One day after isolation, Lipofectamine RNAiMax with siRNA(s) in Optimem was added to a trypsinized islet cell solution to a final concentration of 1,500 cells per 30 µl. Then, 30 µl drops were pipetted onto the lid of a Petri dish. Pseudoislets drops were incubated for 3 days before transfer to conventional Petri dishes with islet medium and incubation for additional 3 days. Pseudoislets were imaged using a Nikon Eclipse Ts2 with NIS-Elements BR 4.51 software.

### Plasma, pancreas and islet hormone content analyses

For quantification of hormones in pancreata, at least half of the fresh pancreas was homogenized in 5 ml ice-cold acid ethanol (1.5% HCl in 75% ethanol). After incubation of the mixture overnight at −20 °C, samples were centrifuged at maximum speed for 2 min, and the supernatant was transferred to a fresh tube. Samples were neutralized with equal amounts of 1 M Tris pH 7.5. Hormones in all samples were quantified using ELISAs: insulin (#90062, Crystal Chem), proinsulin (rat/mouse proinsulin ELISA, #10-1232-01, Mercodia) and glucagon (#10-1281-01, Mercodia).

### GSIS in islets

GSIS studies were performed as recently published^47,48^.

### Ins1E and islet lipidomic analyses

For initial lipidomic analyses of islets from db/db.BKS mice (Fig. 1d,e), islets from eight db/+.BKS (control) and four db/db.BKS mice (from Charles River Laboratories) were pooled, respectively, at 6 and 12 weeks of age. From these, four replicates with 65 islets each were picked, rinsed with PBS and stored as dry pellet at −80 °C until further processing. For lipidomic analyses of islets from ob/ob.B6 and Akita mice, 60 islets per mouse were collected. For d7-sphinganine treatment of db/db.BKS islets, the following protocol was used. After isolation of islets from nine control and ten db/db.BKS mice, islets were cultured overnight in full islet medium. On the morning of the next day, islets were pooled, respectively, and three replicates of 60 islets per timepoint were cultured in SL precursor-depleted (SLPD) medium for approximately 2 h. SLPD medium consisted of DMEM without glucose, glutamine, serine, glycine and sodium pyruvate (Biomol, #D9802-01.10), with addition of 2 mM sodium pyruvate, 44 mM sodium bicarbonate, 11 mM glucose, 0.4 mM glycine, 10% FCS (Gibco OneShot Fetal Bovine Serum, dialysed, Gibco, #A3382001), 100 U ml^−1^ penicillin–streptomycin, as well as GlutaMax, HEPES and β-mercaptoethanol. After the fasting period, islets were cultured in SLPD medium with 1 µM d7-sphinganine (Sigma-Aldrich, #860658 P) for the indicated time periods, followed by washing in PBS and collection. For lipidomic analyses of CerS2^ΔBKO^ islets, 100 islets from islet pools of control and CerS2^ΔBKO^ mice were collected in four independent experiments.

For lipidomic analyses of Ins1E cells, 6 million cells were seeded per sample and collected 48 h later. Lipid amounts were normalized to cell pellet weights. For lipidomic analyses of CerS2:Sgpl1^ΔIns1E^ and control cells, 0.5 million cells were used per sample.

Lipid extraction was performed as described previously^49^. Liquid chromatography was done according to a published protocol^50^ with optimizations. The liquid chromatography was coupled to a hybrid quadrupole-Orbitrap mass spectrometer Q-Exactive (Thermo Fisher Scientific, running Thermo Scientific Q Exactive System Software), samples were analysed in positive and negative mode using a heated electrospray ionization interface. The following parameters were used: spray voltage 3.5 kV, vaporizer temperature of 300 °C, sheath gas pressure 20 AU, aux gas 8 AU and capillary temperature of 320 °C. The detector was set to an MS^2^ method using a data-dependent acquisition (DDA) with top-ten approach with stepped collision energy between 25 and 30. A full scan from 220 to 3,000 *m*/*z* at a resolution of 70,000 was used, while the resolution for MS^2^ was 17,500. A dynamic exclusion filter was applied that excludes fragmentation of the same ions for 20 s. Identification criteria were: (1) resolution with an accuracy of 5 ppm from the predicted mass at a resolving power of 140,000 at 200 *m*/*z*; (2) isotopic pattern fitting to expected isotopic distribution; (3) matching retention time to synthetic standards (if available) and to the in-house database; (4) fragmentation pattern matching the in-house validated fragmentation database. Quantification of lipids were achieved by comparison with the respective internal standard using a one-point calibration. Pooled samples in five concentrations were used as quality controls. Mass spectrometric data analysis was performed in Tracefinder software 4.1 (Thermo Fisher Scientific) for peak picking, annotation and matching to the in-house fragmentation database.

Lipids that could not be detected in all samples were excluded from statistical analyses for volcano plots. For statistical analysis of the lipidome of Sgpl^ΔIns1E^ cells, only SL species were considered. For visualization in volcano plots, lipid amounts were log_2_ transformed and statistical significance was calculated via two-sample equal variance *t*-tests. Lipids with a significant (Benjamini–Hochberg FDR ≤0.05) log_2_ fold change ≥ 1 were regarded as differentially regulated.

### Targeted plasma and Ins1E SL analysis

Ceramides were extracted from murine plasma according to a previously described protocol^51^ and analysed by liquid chromatography with tandem mass spectrometry (LC–MS/MS) as described previously^52^.

For ceramide analyses of CerS1:Sgpl1^ΔIns1E^ and control cells, 4 million cells were seeded per sample. Twenty-four hours later, cells were collected, internal standards (50 µl Sph mix ((50 ng) d17:1 (Avanti LM2000) + (50 ng) d17:0 (Avanti LM2001)), and 50 µl (100 ng) Cer C17 (Avanti 860517), 50 µl (500 ng) DAG d517:0 (Avanti 110538)) were added to all samples, and lipids were extracted as already described^52^.

### TLC

Thin-layer chromatography (TLC) analyses in Ins1E cells were performed using standard methods^53^.

### Islet electron microscopy analyses

Fifty isolated islets from control and CerS2^ΔBKO^ mice, respectively, were pooled and incubated for 6 h in BSA-containing islet medium after overnight regeneration. Islets were washed with BSA-free Krebs-Ringer-Hepes (KRH) buffer and processed, as described previously^48^. Specimens were dehydrated in graded ethanols and embedded in epoxy resin^54^. Ultrathin sections were picked up onto Formvar-carbon-coated grids, stained with lead citrate and viewed under a transmission electron microscope (TEM 910; Zeiss Elektronenmikroskopie, imaging acquisition by iTEM software version 5.2, Olympus Soft Imaging Solutions GmbH). Morphometric evaluation of insulin containing vesicles in β-cells was done using ImageJ.

### Cell culture and generation of knockout cell lines

No cell lines used in this study were found in the database of commonly misidentified cell lines (maintained by ICLAC and NCBI Biosample). Ins1E cells were regularly tested negative for mycoplasma contamination (Eurofins) and their identity confirmed by functional insulin secretion assays, qPCR, immunofluorescence stainings and western blots for β-cell marker mRNAs/proteins. Ins1E cells (including knockout cell lines) were cultivated in RPMI 1640 with 10% FCS, 2 mM Glutamax, 10 mM HEPES, 2 mM Na-pyruvate, 0.18 mM 2-mercaptoethanol and 100 U ml^−1^ penicillin–streptomycin (all Thermo Fisher). CRISPR knockout lines were engineered according to a double single guide RNA (sgRNA) strategy^29^ using the PX459 plasmid or a modified PX458 plasmid^55^. pSpCas9(BB)−2A-GFP (PX458) and pSpCas9(BB)−2A-Puro (PX459) were a gift from Feng Zhang (Addgene plasmids #48138 and #48139). Positive clones were enriched using either puromycin selection or FACS sorting using a FACSAria III (BD Biosciences, running FACSDiva software) or a Cytoflex SRT (Beckman Coulter, running CytExpert software). Monoclonal colonies were grown until near confluency in a 96-well plate, and after passaging, knockout was confirmed using deletion PCR, qPCR and/or immunoblot. At least three monoclonal cell lines were pooled before experiments^56^, if not denoted otherwise in the figure legends.

### Plasmid and siRNA transfections

Transfections were performed using Lipofectamine 2000 #11668019, Life Technologies) or RNAiMax (for siRNA, #13778150, Life Technologies). siRNAs were transfected at final concentrations up to 50 nM.

### ATP content in Ins1E cells

ATP content was measured using the Celltiter-Glo assay (#G7570, Promega) on a Glomax Discover System running System Software V2.4 (Promega) according to the manual. Approximately 1.5 × 10^4^ Ins1E cells were passaged into each well of a 96-well plate 48 h before the Celltiter-Glo assay.

### Proteome analyses of CerS2^∆Ins1E^ cells

Control and CerS2^∆Ins1E^ cells were seeded on p10 dishes in three independent experiments, and collected after 3 h treatment with 0.1% DMSO (for comparison with additional parallel experiments). The weight of each cell pellet was determined and used for normalization. Samples were stored at −80 °C until downstream workup. Dry cell pellets were lysed in denaturating SDS buffer (4% SDS, 100 mM Tris pH 8.0, 100 mM dithiothreitol (DTT), Complete and phosphatase inhibitors 2 and 3 (Sigma-Aldrich); 1:5 (w/v)), by ten strokes through an insulin syringe (needle 26 gauge) followed by sonification (three times for 10s each: 70% pulse for 0.9 s followed by 0.1 s pause). After centrifugation at 75,000*g* for 30 min at 4 °C, supernatants were transferred to fresh reaction tubes and protein concentration was determined by direct photometric measurements (Nanodrop, Thermo Fisher Scientific). To purify and concentrate the sample, a protein equivalent of 10 µg was loaded onto a short SDS–PAGE (10% polyacrylamide, 0.5 cm separation distance as previously described)^57,58^. Subsequently, Coomassie-blue-stained protein bands were excised and subjected to in-gel protease digestion. Therefore, gel slices were alternated washed twice with 25 mM ammonium bicarbonate and 25 mM ammonium bicarbonate and 50% acetonitrile (v/v). Protein reduction was performed in 65 mM DTT for 15 min, shaking at 350 rpm and 50 °C. Subsequent alkylation was done in 216 mM iodacetamide for 15 min in the dark at room temperature. After washing (25 mM ammonium bicarbonate and 25 mM ammonium bicarbonate and 50% acetonitrile (ACN) (v/v)), gel slices were shrinked in 100% ACN. Protein digestion was performed with 400 ng LysC/trypsin mix (Promega) in 25 mM ammonium bicarbonate and 2% ACN (v/v) overnight at 37 °C. Resulting peptides were eluted with 1% trifluoroacetic acid (TFA) (v/v) followed by a second elution with 0.1% TFA/90% ACN (v/v). Peptides were lyophilized and subjected to MS analysis. Hence, lyophilized peptides were reconstituted in 1% TFA (v/v) supplemented with iRT peptides (Biognosys) and separated by liquid chromatography (Ultimate 3000, Thermo Fisher Scientific) using an EASYspray ion source equipped to an Orbitrap Fusion Lumos mass spectrometer (Thermo Fisher Scientific, Thermo Scientific Xcalibur Software, Orbitrap Tune application). Peptides were trapped and desalted on an Acclaim PepMap C18-LC-column (inner diameter (ID): 75 μm, 2 cm length; Thermo Fisher Scientific) and subsequently separated via EASYspray C18 column (ES803; ID: 50 cm × 75 μm inner diameter; Thermo Fisher Scientific) using a 100 min linear gradient from buffer A (0.1% formic acid) to 4–34% buffer B (80% acetonitrile and 0.1% formic acid) at a flow rate of 300 nl min^−1^ followed by a 20 min linear gradient increasing buffer B to 50% and a 1 min linear gradient increasing buffer B to 90%. Column temperature was set to 40 °C. MS data for spectral libraries were acquired in DDA mode. MS spectra were obtained at 120,000 resolution (3 s cycle time), *m*/*z* range of 350–1,600 and a target value of 4 × 10^5^ ions, with maximum injection time of 50 ms. For fragmentation, precursor selection filter was set to charge state between 2 and 7, dynamic exclusion of 30 s and intensity threshold of 2.5 × 10^4^. Fragmentation of precursors was done with an isolation window (*m*/*z*) 1.2, higher-energy collisional dissociation (HCD) energy of 32%, Orbitrap resolution of 15,000 and an ion target value of 1.0 × 10^5^ with maximum injection time of 50 ms. MS data for label free quantification were acquired in the data-independent acquisition (DIA) mode, with each sample run twice. Full-scan MS spectra were obtained at 120,000 resolution, *m*/z range of 400–1,200, and an automatic gain control (AGC) target value of 5 × 10^5^ and maximum injection time of 50 ms. Fragmentation was performed with HCD energy of 32% in 28 windows covering the range from 400 to 1,200 (*m*/*z*) with a segment width of 29.6 (*m*/*z*), Orbitrap resolution of 30,000, AGC target of 1.0 × 10^6^, scan range from 200 to 2,000 (*m*/*z*) and maximal injection time of 60 ms. To calculate protein quantities, single-shot DIA runs were processed in Spectronaut Pulsar (Version 13.12.200217, Biognosys) on the basis of MS^2^ level using factory settings. Spectra were matched against a project-specific spectral library composed out of 44 DDA runs from analogous sample material. The spectral library was generated in Spectronaut Pulsar (Version 12.0.20491.5) from a Proteome Discoverer result file (Version 2.2.0.388; Thermo Fisher Scientific). Used HTSequest search settings were: enzyme trypsin (full), max missed cleavages 2, peptide length 6–144 modifications (carbamidomethyl (C) (fixed), oxidation (M), acetyl (protein N-term) (dynamic)) and FASTA files (*Rattus norvegicus* SwissProt TaxID=10116 (with subtaxonomies, v2017-10-25) and TrEMBL (v2017-10-25)). For label-free quantitative analysis by Spectronaut, identification was done with a *q*-value cut-off of 0.01, matching against the project-specific spectral library (95,235 precursors) and utilizing FASTA file (reviewed SwissProt and Trembl database, rat TaxID 10116 canonical and isoforms, both downloaded from UniProt 07-2018) for Pulsar search. Quantitation was done on MS^2^ level, and area type and global cross-run normalization were performed on median. A two-sided paired *t*-test was performed on precursor ion level, and protein candidates for regulation were filtered by average log_2_ ratio greater than or equal to 0.58 and a BH-FDR less than or equal to 0.05.

### Photoactivatable and clickable sphingosine (PACS)–SILAC SL–protein interactome analysis

Labelling and pull-down of interacting SLs and proteins was performed according to a published protocol^34^ with the following optimizations:

For heavy isotope labelling, the ‘SILAC RPMI Lysine(4) Arginine(6) Kit’ (#284946423) and the ‘SILAC RPMI Lysine(8) Arginine(10) Kit’ (#284986444, Silantes) were used according to the manufacturer’s instructions. To label Sgpl1^∆Ins1E^ cells and CerS2:Sgpl1^∆Ins1E^ cells with stable isotopes, cells were grown in SILAC-RPMI containing 10% dialysed FBS (Silantes), l-glutamine (Silantes), HEPES, penicillin/streptomycin, 2-mercaptoethanol and light (Lys0/Arg0), medium (Lys4/Arg6) or heavy (Lys8/Arg10) amino acids (all Silantes) for at least six passages before the experiment. CerS2:Sgpl1^∆Ins1E^ cells were labelled with light amino acids. Sgpl1^∆Ins1E^ cells were subdivided into two populations. One subpopulation was labelled with medium and the other with heavy amino acids.

For each experiment, 7.5 × 10^6^ light-, medium- and heavy-labelled cells were seeded in 10 cm cell culture dishes, respectively. The next day, cells were washed two times with PBS and treated with 5 µM pacSph (Sigma, #900600 P) in KRH buffer (11 mM glucose) for 1 h at 37 °C and 5% CO_2_. Cells were rinsed three times with ice-cold PBS and covered with ice-cold KRH buffer (11 mM glucose). While heavy-labelled Sgpl1^∆Ins1E^ cells remained in the fridge (−UV), medium-labelled Sgpl1^∆Ins1E^ cells and light-labelled CerS2:Sgpl1^∆Ins1E^ cells were placed on ice and irradiated for 5 min with UV light (Stratagene, Stratalinker 2400, equipped with 3 × 312 nm and 2 × 365 nm tubes) to induce covalent bonds between pacSph-derived SLs and proximal/interacting proteins (+UV). All dishes were rinsed again with ice-cold PBS, before cells were scraped in 1 ml ice-cold PBS, transferred into 1.5 ml reaction tubes and centrifuged at 500*g* for 5 min at 4 °C. Cells were resuspended in 150 µl lysis buffer (PBS with 0.1% SDS, 1% Triton X-100 and 1× protease inhibitor cocktail M (Serva, #39102.01) and incubated for 15 min on ice followed by 15 min on a cell disruptor at 2,500 rpm (Scientific Industries, Digital Disruptor Genie, SI-DD38). After sonication in a water bath sonicator and another 10 min incubation on ice, samples were centrifuged at 14,000*g* for 5 min at 4 °C and the supernatant was transferred in a new reaction tube. Protein concentration was determined via BCA assay (Sigma-Aldrich, #BCA1-1KT).

Two-hundred micrograms of light-, medium- and heavy-labelled protein, respectively, were combined and brought to a volume of 540 µl with lysis buffer. Next, crosslinked pacSph-derived SL/protein complexes were coupled to biotin via click chemistry. To this end, 60 µl click master mix (12 µl 50 mM CuSO4 (Jena Bioscience, #CLK-MI004-50), 30 µl 2 mM TBTA (Sigma, #678937), 12 µl 50 mM ascorbic acid (Sigma, #33034) and 6 µl 10 mM Picolyl.azide-PEG4.Biotin (Jena Bioscience, #CLK-1167-5)) was added to the combined proteins, followed by an incubation for 90 min at 37 °C under constant shaking at 900 rpm. Next, the proteins were precipitated via methanol/chloroform to remove unclicked biotin-azide. In brief, 2,400 µl ice-cold methanol, 600 µl ice-cold chloroform and 1,800 µl ice-cold ddH_2_O were added to the proteins and mixed vigorously, before centrifugation for 15 min at 2,800*g* and 4 °C. The upper phase was discarded and the proteins were pelleted by adding 2,400 µl ice-cold methanol followed by centrifugation for 5 min at 2,800*g* and 4 °C. The supernatant was discarded and the pellet was resuspended in 300 µl 2% SDS/PBS. After sonication in a water bath sonicator, proteins were precipitated a second time by adding 1,200 µl ice-cold methanol, 300 µl ice-cold chloroform and 900 µl ice-cold ddH_2_O, followed by vigorous mixing and centrifugation for 15 min at 2,800*g* and 4 °C. The upper phase was discarded, 1,200 µl ice-cold methanol was added and the proteins were pelleted for 5 min at 14,000*g* and 4 °C. The supernatant was discarded and the protein pellet was air dried for 10 min at room temperature. To resuspend the proteins, 50 µl 4% SDS/PBS was added to the pellet, followed by a 30 min incubation at 37 °C and constant shaking at 900 rpm. Afterwards, the sample was diluted to 2% SDS with PBS before incubation at 37 °C and 900 rpm for further 20 min. Next, the sample was diluted to 0.2% SDS and proteins were incubated for another 10 min at 37 °C and 900 rpm. Resuspension of the protein pellet was supported by manual pipetting every 15–20 min and sonication in a water bath sonicator after the dilution step to 2% SDS. Insoluble precipitates were pelleted by centrifugation for 1 min at 800*g*, and just the supernatant was used for further processing.

To pull down the biotin–SL–protein complexes, 180 µl streptavidin magnetic beads (NEB, S1420S) were first equilibrated for 5 min in equilibration buffer (50 mM Tris pH 7.4, 150 mM NaCl, 0.05% Triton X-100 and 1 mM DTT) and second for 5 min in 0.2% SDS/PBS. Afterwards, beads were combined with the resuspended proteins and incubated overnight at 4 °C on an orbital shaker. Beads were rinsed 17 times with 1% SDS/PBS and 3 times with PBS. To elute bound complexes, beads were incubated with 20 µl elution buffer (10 mM Tris pH 7.4, 2% SDS, 5% 2-mercaptoethanol and 2 mM biotin (Sigma, B4501-1G)) for 10 min at room temperature, followed by an incubation for 15 min at 98 °C. The eluate was separated from the beads and frozen at −80 °C until further processing. A magnetic rack was used for all washing steps.

Eighteen microlitres from each eluate was loaded onto SDS–PAGE (10% polyacrylamide, 0.5 cm separation distance) and subjected to in-gel protein digestion as previously described^57,58^. Digestion was performed with 400 ng LysC/Trypsin Mix (Promega) in 25 mM ammonium bicarbonate and 2% (v/v) ACN overnight at 37 °C. Resulting peptides were eluted first with 1% (v/v) TFA followed by elution with 0.1% TFA/90% (v/v) ACN and lyophilization. For MS analysis, lyophilised peptides were reconstituted in 1% TFA (v/v) and separated by liquid chromatography (Ultimate 3000, Thermo Fisher Scientific). Peptides were trapped and desalted on an Acclaim PepMap C18-LC-column (ID: 75 μm, 2 cm length; Thermo Fisher Scientific) and subsequently separated via an Aurora C18 column (AUR2-25075C18A, 25 cm × 75 μm C18 1.6 µm; IonOpticks) using a 2 h three-step gradient at a total flow rate of 300 nl min^−1^ with buffer A (0.1% formic acid) and buffer B (80% ACN and 0.1% formic acid). First for 72 min linear from 2% to 19% buffer B, second for 28 min from 19% to 29% buffer B, followed by 20 min from 29% to 41% buffer B and a 1 min linear gradient increasing buffer B to 95%.

Ionization of peptides was done within Nanospray Flex source (Thermo Fisher Scientific) equipped with a column oven (PRSO-V2, Sonation lab solution), set to 40 °C. MS spectra were obtained on an Orbitrap Exploris 480 mass spectrometer (OE480, Thermo Fisher Scientific, Thermo Scientific Xcalibur Software, Orbitrap Tune application) with 240,000 resolution, *m*/*z* range of 350–1,500 and an AGC target value of 300%, with auto mode maximum injection time and a cycle time of 2 s. For MS2 spectra, acquisition precursor selection filter was set to charge state between 2 and 6, dynamic exclusion of 45 s and mass tolerance of 10 ppm. To account for stable isotope labelling by amino acids in cell culture (SILAC), target masses were selected (three precursors in a group) with label-specific mass differences: 4.025 Lys-4 (4D), 8.014 Lys-8 (6C132N15), 6.02 Arg6 (6C13) and 10.008 Arg-10(6C134N15) and a group intensity range from 2% to 100% of the most intense precursor. The most intense ion of a triplet with an intensity threshold of 1 × 10^4^ was selected. Fragmentation of filtered precursors was done with an isolation window (*m*/*z*) 1.6, HCD energy of 30%, Orbitrap resolution of 7,500 and an AGC target value of 100% with maximum injection time of 36 ms. For bioinformatical analyses, raw files were searched with MaxQuant 1.6.17.0 (www.maxquant.org)^59^ against an UniProt Reference Proteome database specific for *Rattus norvegicus* (release 2020_11, 29,942 entries) and against MaxQuant’s internal common contaminants database. Enzyme specificity was set to Trypsin/P with up to two missed cleavages allowed and a minimal peptide length of 7. Quantification was set to SILAC 3plex with Arg6 and Lys4 as medium and Arg10 and Lys8 as heavy label. Regarding modifications, carbamidomethyl at cysteine was selected as fixed and oxidation of methionine, acetylation of protein N-termini and heavy-labelled proline (Pro5 and Pro6) were selected as variable modifications. Mass tolerances were 20/4.5 ppm for the first/main search at the MS1 and 20 ppm at the MS2 level. The ‘match between run’ and the ‘re-quantify’ options were enabled using default parameters. Identifications were filtered at an FDR threshold of ≤1% at the peptide and protein level using a reverse decoy approach. Protein quantification was calculated using unique and razor peptides and included peptides with the following modifications: Oxidation (M), Acetyl (Protein N-term), Pro5 and Pro6. The minimal ratio count was set to 2, and advanced ratio estimation was enabled. The resulting proteinGroups table was further analysed using Perseus 1.6.15.0 (https://www.maxquant.org/perseus/)^60^ together with plugins linking to R 3.6.3 (https://www.R-project.org/). First reverse, contaminant and identified by site hits were removed. Resulting protein intensities were log_2_ transformed, and all proteins having less than two valid values in one of the UV-treated samples were removed. To determine specific interactors for SLs, ratios against the non-UV control were calculated for both UV-treated samples (Sgpl1^∆Ins1^ +UV/Sgpl1^∆Ins1E^ cells −UV, CerS2:Sgpl1^∆Ins1E^ cells +UV/ Sgpl1^∆Ins1E^ cells −UV). After one-sample *t*-tests against 0, proteins exhibiting a significant (Benjamini–Hochberg FDR ≤0.05) log_2_ fold change ≥1 were considered true interactors. In addition, proteins exclusively present in UV+ samples were also considered. In the next step, to assess differences in SL interactors between Sgpl1^∆Ins1E^ cells and CerS2:Sgpl1^∆Ins1E^ cells samples, missing intensity values were imputed (from normal distribution, width: 0.3, down shift: 1.8, mode: separate for each column) and the data normalized using both the ‘Remove batch effect’ (Method: limma) and the ‘Quantile normalization’ function. Finally, two-sample *t*-tests were performed to identify significantly different proteins (Benjamini–Hochberg FDR ≤0.05, log_2_ fold change ≥0.58). Moreover, proteins exclusively present in one of the two conditions were also considered.

GO-term enrichment analyses were performed via PANTHER overrepresentation test (http://www.geneontology.org, http://pantherdb.org, GO Ontology database DOI: 10.5281/zenodo.6799722, released 2022-07-01) using Fisher’s exact test with FDR correction.

### Experimental verification of SL protein interactions

For experimental verification of SL protein interactions with transient overexpression, approximately 7.5 × 10^5^ cells were lipofected with plasmids coding for Myc-DDK-tagged versions of putative interactors (Fxyd6 (Origene: RC210607), Bst2 (Origene: RC207540) and Tmed1 (Origene: RC200255)). Twenty-four hours later, cells were pulsed with 5 µM pacSph and UV irradiated, followed by click-chemistry-based coupling of biotin and pull-down. For experimental verification of endogenous SL–protein interactions, approximately 4 × 10^6^ cells were used per sample.

For data analysis, band intensities (background corrected) were determined using the ImageLab software (Bio-Rad). For data analysis, band intensities (background corrected) were determined using the ImageLab software (Bio-Rad). Intensities from eluate bands were normalized to the intensities from the respective input band.

### qPCR analyses

qPCR analyses were performed as published^61^ using TRIzol (#15596026, Thermo Fisher) or RNA-Easy Kits (Qiagen), High-Capacity cDNA Kits (Thermo Fisher, 4368814) and Perfecta SybrGreen mastermix (#733-1250, QuantaBio) or Luna Universal qPCR Master Mix (#M3003E, New England Biolabs) on a QuantStudio 7 running QuantStudio 7 Flex System Software V1.3 (Thermo Fisher Scientific). Gene expression was analysed using the ddCt method. 36b4 and/or Gusb were used as housekeeper mRNAs.

### Immunoblot analyses

Immunoblot analyses were performed using standard methods^61^ using pre-made gels (Mini-PROTEAN TGX Stain-Free Precast Gels; Bio-Rad). Protein amount per lane was detected by a proprietary trihalo compound, which is contained in stain-free gels (and transferred to the membrane, Bio-Rad). This signal was used for normalization of all bands of interest if not stated otherwise. Quantification was performed using Image Lab software (Bio-Rad) after imaging on a Chemidoc MP running System Software 5.2.1 (Bio-Rad).

### Antibodies, siRNAs, sgRNAs and primers

Antibodies (with dilutions), siRNAs, sgRNAs and primer information are presented in Supplementary Table 13.

### Flow cytometric assays

For proliferation analysis, the 5-ethynyl-2′-deoxyuridine (EdU) assay was performed according to the manufacturer’s instructions (#C10425, Thermo Fisher) with 5 µM EdU treatment for 2.5 h. For assessment of viability (for example, after pacSph treatment), propidium iodide (PI)-positive and PI-negative cells were counted by flow cytometry as described^61^. For quantification of β-cell granularity, islets were isolated from control and Cers2^ΔBKO^ mice, expressing TdTomato^45^ Cre-dependently. Granularity was assessed using the side-scatter area (SSC-A). Cells were recorded either on a FACSCalibur (BD Biosciences running CellQuest Pro software) for analysis of EdU incorporation and viability, or on a Cytoflex S (Beckman Coulter, running CytExpert system software) for analysis of granularity. Data were quantified using FlowJo V10 software (BD Biosciences). Gating strategies are shown in Supplementary Fig. 4.

### ER area quantification in Ins1E cells

Imaging was performed on an Operetta CLS high-content screening microscope (PerkinElmer, running Harmony V4 software) with a 40× air objective in spinning disc confocal mode. Each staining was performed in three wells, and we recorded images for 16 sites per well. For each site, the images of a single focal plane were segmented with a CellProfiler (version 4.1.3) routine^62^. We detected the nuclei and ER signals via image segmentation and used the nuclei masks as a seed for the determination of the cell area by an expansion by 25 pixels. Various morphometric measures were determined for the detected objects. ER area measurements were normalized to the cell area measurements. The numeric data were pre-processed using KNIME (version 4.3) before visualization and analysis^63^.

### Human islet RNA-seq

Published human islet RNA-seq^26^ was re-analysed with Salmon using Gencode 38 as reference as described^64^. Bulk RNA-seq datasets were downloaded from GEO under accession number GSE152615 (ref. ^41^) for brefeldin-A-exposed human islets (0.1 μg ml^−1^ for 24 h) and under GSE159984 (ref. ^65^) for human islets exposed to palmitate (0.5 mM) and/or glucose (22.2 mM) for 48 h. For each dataset, raw sequencing reads were processed with fastp 0.19.6 (ref. ^66^) using the default parameters for quality control, adaptor trimming and low-quality filtering to obtain clean reads. Gene expression was quantified by Salmon 1.4.0 (ref. ^67^) with parameters ‘–seqBias–gcBias–validateMapings’ using GENCODE v36 (GRCh38.p13) as the genome reference and normalized as transcripts per million. Differential gene expression was assessed with R package DESeq2 1.28.1 (ref. ^68^). Log_2_ fold change and Benjamini–Hochberg-corrected *P* values were computed by the Wald test of the DESeq2 algorithm.

### Automated Ins1E cell counts

Staining and quantification of CerS2^ΔIns1E^ cells was performed as published^61^. For adenovirus experiments, cells were imaged on a Cytation 5 (BioTek) and counted using Cytation 5 Gen5 standard software.

### TMRM-based determination of mitochondrial membrane potential

Cells were seeded on 35 mm glass-bottom dishes (MatTek) coated with poly-d-lysine (0.1 mg ml^−1^) (Sigma-Aldrich). The next day, cells were stained for 30 min with 100 nM mitotracker green (MTG) (Invitrogen) and 50 nM tetramethylrhodamine methyl ester perchlorate (TMRM) (Sigma-Aldrich), and then washed three times with corresponding cell culture media followed by imaging of the respective samples. The measurement of mitochondrial membrane potential was performed using a Perkin Elmer Spinning Disc microscope equipped with a 60× oil-immersion objective (numerical aperture 1.49) and a Hamamatsu C9100 camera using its standard software. Cells were maintained at 37 °C and 5% CO_2_ while imaging. For each field of view, 20 optical sections were imaged where each optical section had a step size of 0.6 µm, which was sufficient to cover the thickness of the whole cell. TMRM and MTG were excited using 560 and 488 nm laser lines, respectively. The corresponding emission filters, used for collecting TMRM and MTG intensities, were 615 ± 35 nm and 527 ± 27.5 nm. Image analysis was performed using Fiji software. After background subtraction and maximum-intensity *Z* projection, regions of interest encompassing individual cells were marked using the polygon tool in Fiji, followed by determining the ratio of mean TMRM to MTG signal intensities per cell.

### Co-immunoprecipitation of Tmed2-V5 and Pro-/Pcsk1-DDK

A plasmid expressing human full-length Tmed2 (with a C-terminal linker (amino acid sequence TRTRPL) followed by a V5 tag (amino acid sequence GKPIPNPLLGLDST)) under control of a Rous-sarcoma virus promoter was synthesized (Integrated DNA Technologies). As both N- and C-terminal regions of Pcsk1 are cleaved during maturation, we co-expressed a DDK-tagged (Pro-)Pcsk1 protein in which the DDK tag was inserted behind the N-terminal cleavage sites, also allowing for detection of mature Pcsk1 (refs. ^69,70^). As determined in optimization experiments, a 5:1 ratio of Pcsk1-DDK plasmid to Tmed2-V5 plasmid was used for the transfection for the co-immunoprecipitation experiments. Samples were collected 24 h after lipofection, and protein was immunoprecipitated using anti-FLAG M2 magnetic beads (Merck, #M8823) on a magnetic rack.

### Co-localization experiments

Tmed2-V5 and Pro-/Pcsk1-DDK expression plasmids were lipofected in 96-well format and fixed 24 h later. Nuclei were counterstained using Sytox Green (Thermo Fisher, #S7020). Imaging was performed on a LSM 880 (Zeiss, running various versions of Zen Black) confocal microscope using a 63× objective in confocal mode. Overlap of Tmed2-V5 and Pro-/Pcsk1-DDK in at least ten cells per genotype per each of two independent experiments was quantified using CellProfiler. To determine overlap of Tmed2 and Pcsk1 with ER and Golgi, we used the following plasmids: Tmed2-Myc-DDK (Origene, #RC206849), Pcsk1-Myc-DDK (DDK-tag will be cleaved off during maturation into enzymatically active Pcsk1; Origene, #MR225451) and the Pro-/Pcsk1-DDK plasmid described above. Following staining, imaging was performed with an Operetta CLS high-content screening microscope (PerkinElmer, running Harmony V4 software) with a 40× air objective in spinning disc confocal mode. Nuclei, DDK and V5 signals were segmented by minimal cross-entropy algorithms. The cell areas were detected by expanding the nuclei objects again using a minimal cross-entropy algorithm and using the broader cell staining (for example, protein disulfide isomerase (PDI) signal) as a basis to define the cell limits. Subtraction of the nuclei from the cell objects finally resulted in the identification of the cytoplasm areas. Subsequently, the objects were related and overlaps were measured using the MeasureObjectOverlap module of CellProfiler.

### Adenoviral overexpression of CerS6

Ins1E cells were infected with Ad-Control (VQAd CMV eGFP, Viraquest) and Ad-hCerS6 (Vector Biolabs, #ADV-213700) in full medium at indicated multiplicity of infection for 6 h, followed by medium exchange and collection of the cells 36 h later.

### Statistics and reproducibility

No statistical method was used to pre-determine sample size, but it was based on preliminary data and previous publications as well as observed effect sizes. Experiments were not randomized owing to concurrent use of several genotypes and treatments. Investigators were not blinded to allocation of genotypes and treatments. Data were excluded only when positive controls failed, obvious technical issues occurred or animals were killed in line with local animal guidelines. Values are reported as mean ± standard error of the mean (s.e.m.) unless stated otherwise. Data are derived from at least two independent experiments, unless stated otherwise. Each statistical test is described in the figure legends. Mixed-effects analysis was used instead of two-way analysis of variance (ANOVA), when data points were missing (for example, owing to euthanization of animals). All Student’s *t*-tests were unpaired and two-tailed unless stated otherwise. We used various versions of Excel and Powerpoint (both Microsoft), Photoshop and Illustrator (both Adobe) for data collection and generation of figures; and Graphpad Prism Software (Version 7, 8 or 9) and RStudio (version 1.2.5001) with the packages ‘dplyr’, ‘ggplot2’, ‘ggrepel’, ‘IHW’ and ‘matrixTest’ for statistical analysis and data visualization, unless stated otherwise.

### Reporting summary

Further information on research design is available in the Nature Portfolio Reporting Summary linked to this article.

## Online content

Any methods, additional references, Nature Portfolio reporting summaries, source data, extended data, supplementary information, acknowledgements, peer review information; details of author contributions and competing interests; and statements of data and code availability are available at 10.1038/s41556-022-01027-2.

## Supplementary information

Supplementary Information Supplementary Figs. 1–4, figure legends for Supplementary Figs. 1–4 and source blots for Supplementary Figs. 1–4.

Reporting Summary

Supplementary Table 1 Supplementary Table 1. Lipidomics raw data of islets from 6- and 12-week-old db/db and db/+ mice. Supplementary Table 2. Calculations based on lipidomics raw data of islets from 6- and 12-week-old db/db and db/+ mice for generation of volcano plots. Supplementary Table 3. Lipidomics raw data of islets from 7-week-old Akita and control mice. Supplementary Table 4. Lipidomics raw data of islets from 12-week-old ob/ob and control mice. Supplementary Table 5. Lipidomics raw data of d7-sphinganine incorporation in islets from 12-week-old db/db and db/+ mice. Supplementary Table 6. Calculations based on lipidomics raw data of the d7-sphinganine incorporation experiment in islets from 12-week-old db/db and db/+ mice for generation of volcano plot. Supplementary Table 7. Proteomics data of control versus Cers2^ΔIns1E^ cells. Supplementary Table 8. Lipidomics raw data of Sgpl^ΔIns1E^ and Sgpl:Cers2^ΔIns1E^ cells treated with 5 µM pacSph or solvent control (EtOH). Supplementary Figure 9. Chemoproteomic screen to identify SBPs in Ins1E cells. Comparison of Sgpl^ΔIns1E^ +UV (control+) vs. Sgpl^ΔIns1E^ −UV (control−). Supplementary Table 10. Chemoproteomic screen to identify SBPs in Ins1E cells. Comparison of Sgpl:CerS2^ΔIns1E^ +UV (Cers2KO) versus Sgpl^ΔIns1E^ −UV (control−). Supplementary Table 11. Chemoproteomic screen to identify CerS2-dependent SBPs in Ins1E cells. Comparison of Sgpl:CerS2^ΔIns1E^ +UV (Cers2KO) versus Sgpl^ΔIns1E^ +UV (control+). Supplementary Table 12. Chemoproteomic screen to identify CerS2-dependent SBPs in Ins1E cells. Comparison of Sgpl:CerS2^ΔIns1E^ +UV (Cers2KO) versus Sgpl^ΔIns1E^ +UV (control+). Proteins found exclusively in Sgpl:CerS2^ΔIns1E^ +UV (Cers2KO). Supplementary Table 13. List of sgRNAs, primers and antibodies used in this study.

Supplementary Data 1 Source numerical data for supplementary figures.

## Data Availability

MS data have been deposited in ProteomeXchange^71^ via the PRIDE^72^ partner repository with the dataset identifiers PXD029848 and PXD029781. Akita, ob/ob and db/db.BKS (sphingo-) lipidome datasets are presented in Supplementary Tables 1–6; CerS2^ΔIns1E^ cell proteome data are presented in Supplementary Table 7; the pacSph incorporation dataset is presented in Supplementary Table 8; identified SBPs (CerS2 dependent and independent) are presented in Supplementary Tables 9–12; sgRNA, siRNA, primer and antibody information is presented in Supplementary Table 13. All other data supporting the findings of this study are available from the corresponding author on reasonable request. Source data are provided with this paper.

## Source data

Source Data Fig. 1 Statistical source data.
Source Data Fig. 2 Statistical source data.
Source Data Fig. 3 Statistical source data.
Source Data Fig. 4 Statistical source data.
Source Data Fig. 4 Unprocessed western blots.
Source Data Fig. 5 Statistical source data.
Source Data Fig. 5 Unprocessed western blots.
Source Data Extended Data Fig. 1 Statistical source data.
Source Data Extended Data Fig. 2 Statistical source data.
Source Data Extended Data Fig. 3 Statistical source data.
Source Data Extended Data Fig. 4 Statistical source data.
Source Data Extended Data Fig. 4 Unprocessed western blots.
Source Data Extended Data Fig. 5 Statistical source data.
Source Data Extended Data Fig. 5 Unprocessed western blots.
Source Data Extended Data Fig. 6 Statistical source data.
Source Data Extended Data Fig. 6 Unprocessed TLC image.
Source Data Extended Data Fig. 7 Statistical source data.
Source Data Extended Data Fig. 7 Unprocessed western blots.
Source Data Extended Data Fig. 8 Statistical source data.
Source Data Extended Data Fig. 8 Unprocessed western blot.
Source Data Extended Data Fig. 9 Statistical source data.
Source Data Extended Data Fig. 9 Unprocessed western blot.
Source Data Extended Data Fig. 10 Statistical source data.
Source Data Extended Data Fig. 10 Unprocessed western blots.
