# Supplementary material for: Sphingolipid subtypes differentially control proinsulin processing and systemic glucose homeostasis
Source: Nat Cell Biol. 2022 Dec 21;25(1):20–9. doi: 10.1038/s41556-022-01027-2 (PMC9859757; doi:10.1038/s41556-022-01027-2)

nature cell biology

Letter

https://doi.org/10.1038/s41556-022-01027-2

Sphingolipid subtypes differentially control proinsulin processing and systemic glucose homeostasis

In the format provided by the authors and unedited

Supplementary Figure 1

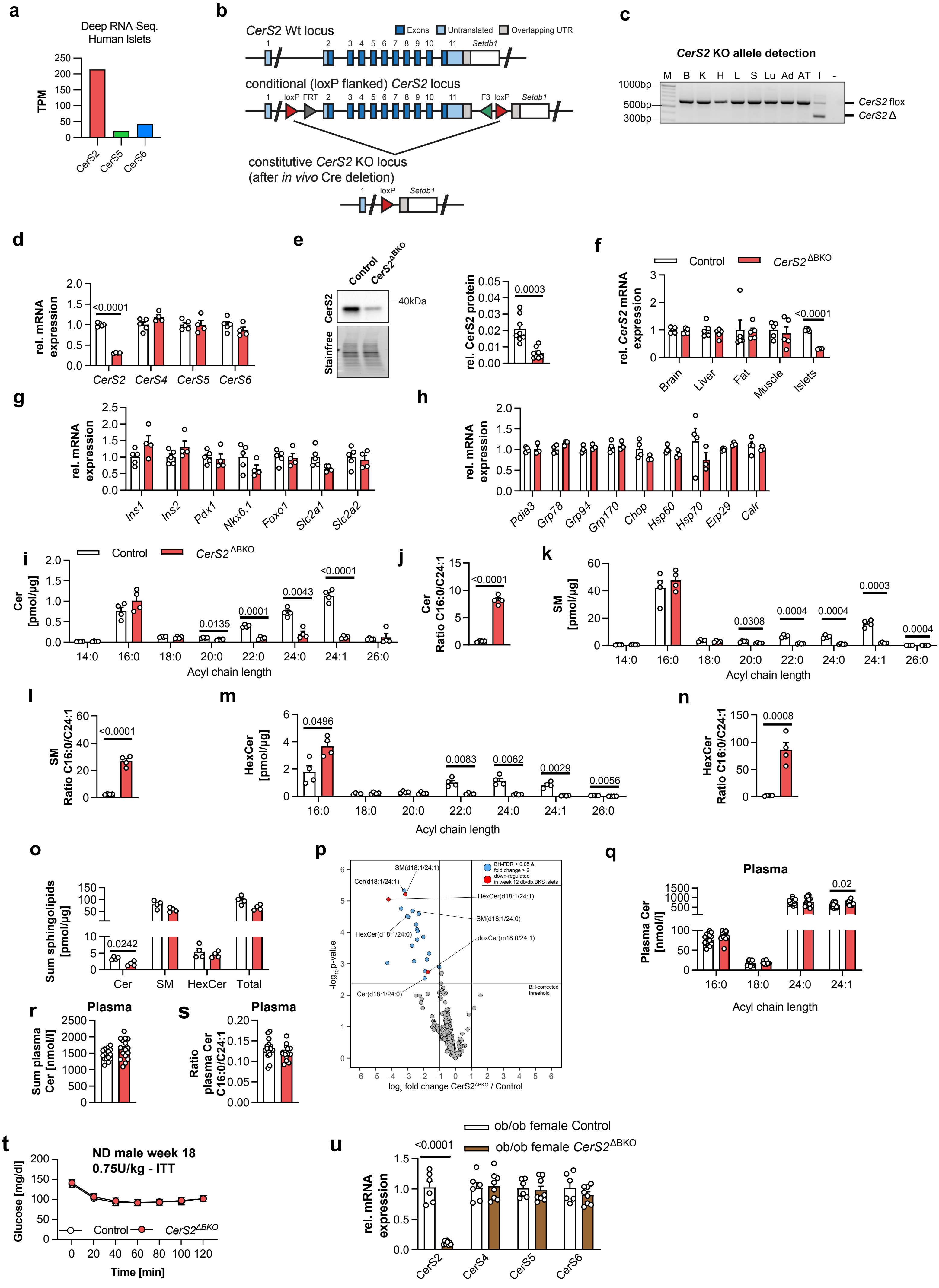

Supplementary Figure 2

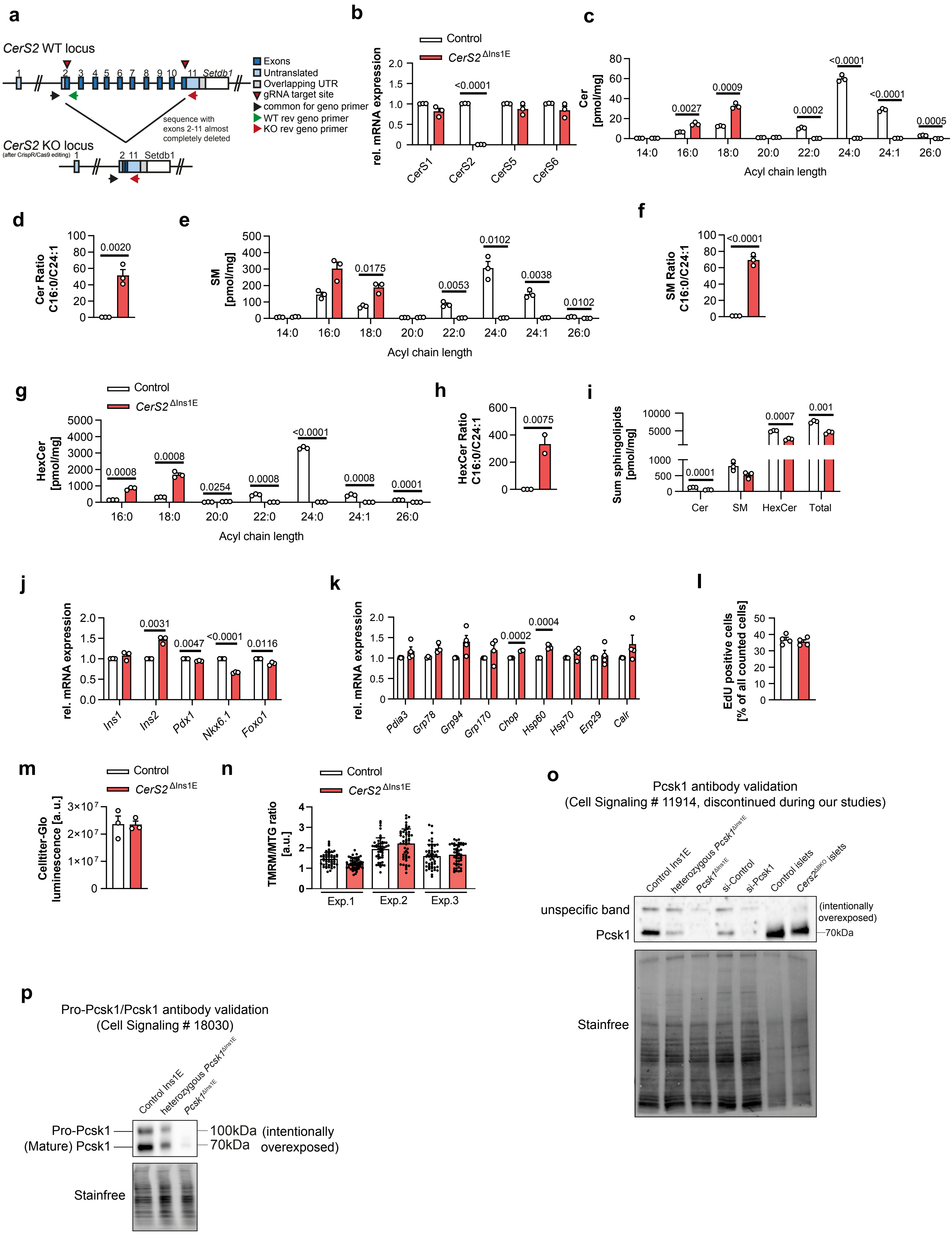

Supplementary Figure 3

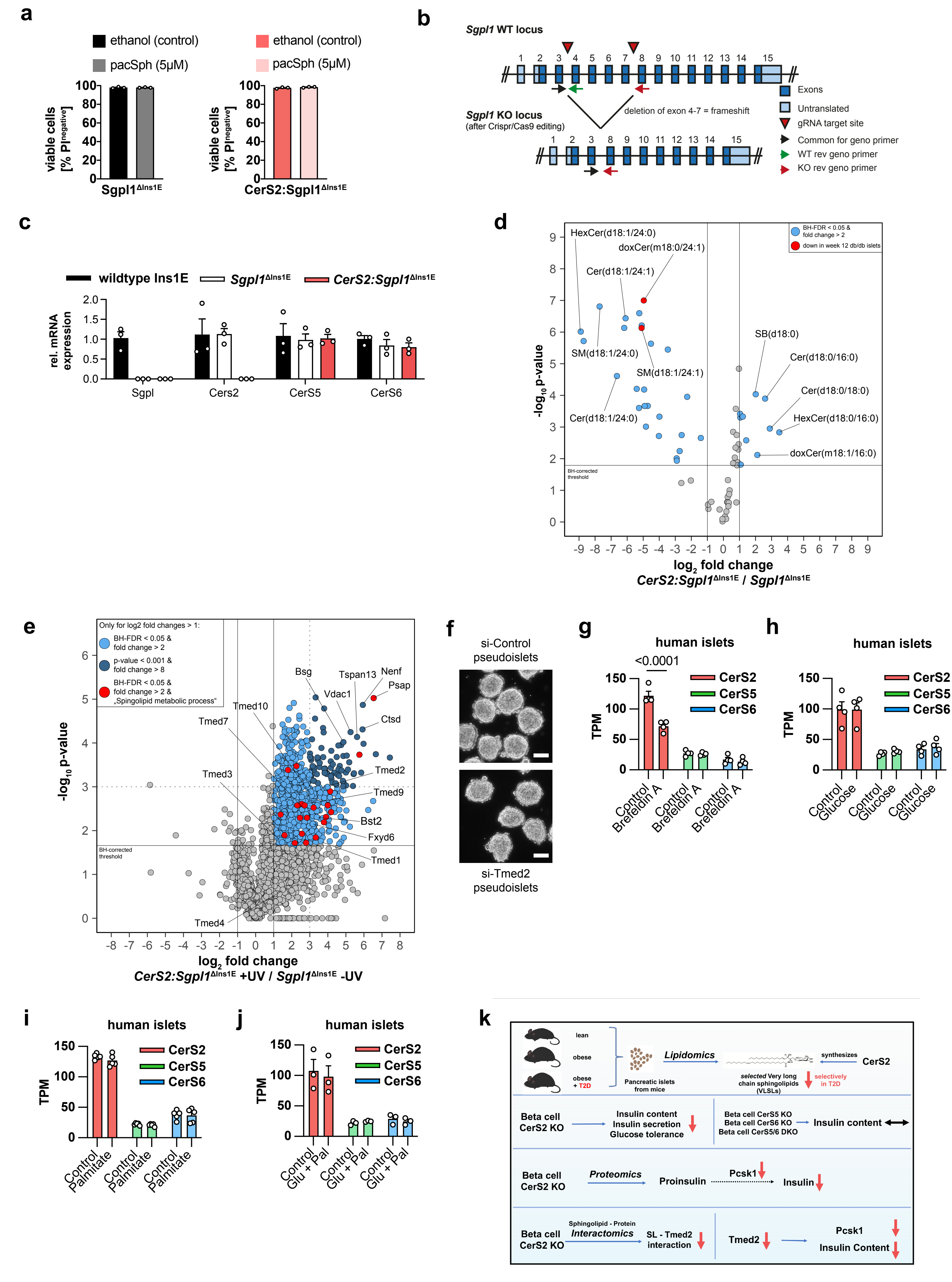

Supplementary Figure 4

A

Gating strategy for granularity assay
(measured on a Cytoflex S flow cytometer (Beckman Coulter))

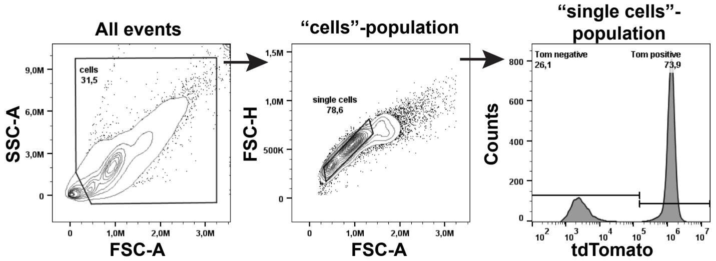

Gating for Extended Data Figure 3i (Tom positive = beta cells)

B

Gating strategy for EdU assay
(measured on a FACS Calibur flow cytometer (BD))

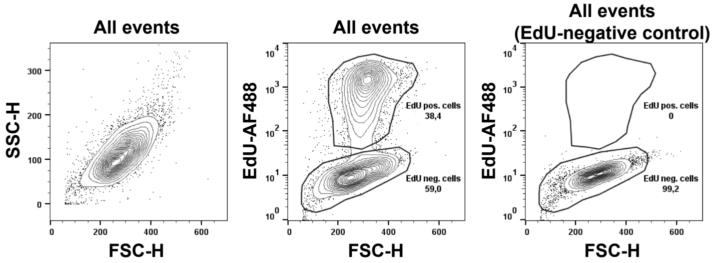

Gating for Supplementary Figure 2l

C

Gating strategy for viability assay
(measured on a FACS Calibur flow cytometer (BD))

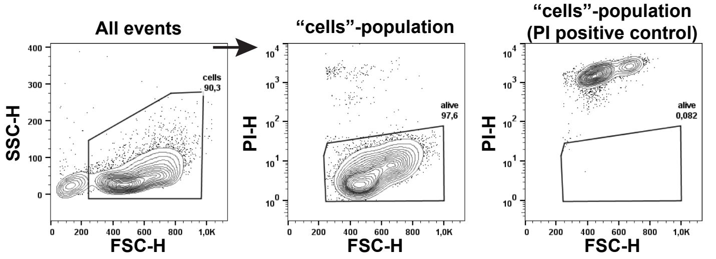

Gating for Supplementary Figure 3a

Supplementary Figure legends

Supplementary Figure 1: Additional phenotyping of CerS2$^{\Delta BKO}$ mice.

a, Expression levels (Transcripts per Kilobase Million, TPM) of protein-coding isoforms of CerS2, -5 and -6 in 5 independent human islet preparations determined by re-analysis of deep RNA-sequencing data. For each CerS, the mean TPMs of each detected protein-coding isoform was added up. Published Data (see methods) was re-analyzed with Salmon using Gencode 38 as reference. **b**, Overview of conditional knockout strategy for CerS2 allele. **c**, Representative PCR result for detection of deleted CerS2 allele in several tissues from one CerS2$^{\Delta BKO}$ mouse. M = marker, B = brain, K = kidney, H = heart, L = liver, Ad = adrenal gland, S = skeletal muscle, Lu = lung, AT = adipose tissue, I = pancreatic islets, "-" = water control. **d**, Relative mRNA expression of various CerS family members in pancreatic islets of adult male control and CerS2$^{\Delta BKO}$ mice (n = 5 control vs. 4 CerS2$^{\Delta BKO}$ mice). **e**, Representative immunoblot (left) and quantification (right) of CerS2 protein levels in islets from adult male control and CerS2$^{\Delta BKO}$ mice (n = 8 control vs. 8 CerS2$^{\Delta BKO}$ mice). Stain-Free signal was used for normalization of immunoblots. **f**, Relative expression levels of CerS2 in various tissues of control and CerS2$^{\Delta BKO}$ mice (n = tissue samples from 5 control vs. 5 CerS2$^{\Delta BKO}$ mice; for islets only samples from 4 CerS2$^{\Delta BKO}$ mice). **g**, Relative beta cell marker mRNA expression in islets of adult male control and CerS2$^{\Delta BKO}$ mice (n = 5 control vs. 4 CerS2$^{\Delta BKO}$ mice). **h**, Relative ER stress and mitochondrial stress marker mRNA expression in islets of adult male control and CerS2$^{\Delta BKO}$ mice (n = 4 control vs. 3 CerS2$^{\Delta BKO}$ mice). **i**, Quantification of specific ceramide species in islets of adult male control and CerS2$^{\Delta BKO}$ mice. **j**, C16:0/C24:1 ceramide ratio in islets of adult male control and CerS2$^{\Delta BKO}$ mice. **k**, Quantification of specific sphingomyelin species in islets of adult male control and CerS2$^{\Delta BKO}$ mice. **l**, C16:0/C24:1 sphingomyelin ratio in islets of adult male control and CerS2$^{\Delta BKO}$ mice. **m**, Quantification of specific hexosylceramide species in islets of adult male control and CerS2$^{\Delta BKO}$ mice. **n**, C16:0/C24:1

hexosylceramide ratio in islets of adult male control and CerS2$^{\Delta BKO}$ mice. **o**, Sum of all detected Cer, SM and HexCer species in islets of adult male control and CerS2$^{\Delta BKO}$ mice. In (j, k, m and o) lipid amounts are normalized to protein content. **p**, Volcano plot showing $\log_2$ fold change of lipids in islets between CerS2$^{\Delta BKO}$ and control mice plotted against the $-\log_{10}$ *p*-value of a two-sided equal variance *t*-test. Log_2 fold change >1 and Benjamini-Hochberg false discovery rate (BH-FDR) <0.05 was used as cut-off for significance. Only lipids detected in all samples were used for calculation. For (i-p) 100 islets from islet pools of control and CerS2$^{\Delta BKO}$ mice were harvested in 4 independent experiments. Lipid species indicated in red were detected as downregulated in islets from obese diabetic mice (see Figure 1). **q**, Quantification of detected specific ceramide species in plasma samples of adult ND-fed male control and CerS2$^{\Delta BKO}$ mice (n = 15 control vs. 15 CerS2$^{\Delta BKO}$ mice). **r**, Sum of all detected ceramide species in plasma of adult male control and CerS2$^{\Delta BKO}$ mice (n = 15 control vs. 16 CerS2$^{\Delta BKO}$ mice). **s**, Ratio of C16:0/C24:1 Cer in plasma of adult male control and CerS2$^{\Delta BKO}$ mice (n = 15 control vs. 15 CerS2$^{\Delta BKO}$ mice). **t**, Results of intraperitoneal insulin tolerance tests (ITT) in ND-fed adult male control and CerS2$^{\Delta BKO}$ mice (n = 16 control vs. 16 CerS2$^{\Delta BKO}$ mice). **u**, Relative mRNA expression of various CerS in islets of adult female ob/ob control and ob/ob CerS2$^{\Delta BKO}$ mice (n = 6 ob/ob control vs. 8 ob/ob CerS2$^{\Delta BKO}$ mice). Statistical analysis was performed using unpaired two-sided Student's *t*-test (e, j, l, n, r, s), or multiple two-sided *t*-tests with Holm-Sidak correction (d, f-i, k, m, o, q, u) or two-way ANOVA with Sidak's multiple comparisons test (t). Data points in (d, f-h, q-s, u) depict samples (islets, plasma) from unique mice. Data points in (i-o) represent individual islet pools. Bar graphs represent means + s.e.m. (d, f-o, q-s, u) or means (a). Data points in (t) represent means ± s.e.m. *p*-values are stated in each figure.

Supplementary Figure 2: CerS2$^{\Delta Ins1E}$ cell phenotyping and Pcsk1 antibody validation.

a, Double cut CrispR/Cas9 CerS2 knockout strategy in Ins1E cells. **b**, Relative mRNA expression of various CerS in control and CerS2$^{\Delta Ins1E}$ cells (*n* = 3 independent experiments). **c**, Lipidomic analysis of Cer species in control and CerS2$^{\Delta Ins1E}$ cells. **d**, Ratio of C16:0/C24:1 Cer in control and CerS2$^{\Delta Ins1E}$ cells. **e**, Lipidomic analysis of SM species in control and CerS2$^{\Delta Ins1E}$ cells. **f**, Ratio of C16:0/C24:1 SM in control and CerS2$^{\Delta Ins1E}$ cells. **g**, Lipidomic analysis of HexCer species in control and CerS2$^{\Delta Ins1E}$ cells. **h**, Ratio of C16:0/C24:1 HexCer in control and CerS2$^{\Delta Ins1E}$ cells. **i**, Sum of all detected Cer, SM and HexCer species in control and CerS2$^{\Delta Ins1E}$ cells. For (c-i), *n* = 3 independent experiments. In (c, e, g and i) lipid amounts are normalized to cell pellet weights. **j**, Relative expression of beta cell markers in control and CerS2$^{\Delta Ins1E}$ cells (*n* = 3 independent experiments). **k**, Relative expression of ER and mitochondrial stress markers in control and CerS2$^{\Delta Ins1E}$ cells (*n* = 4 independent experiments, except for Grp78 and Chop *n* = 3 independent experiments). **l**, EdU incorporation as a marker of proliferation rates in control and CerS2$^{\Delta Ins1E}$ cells (*n* = 4 independent experiments). **m**, Cell-Titer Glo luminescence as a marker of ATP content in control and CerS2$^{\Delta Ins1E}$ cells (*n* = 3 independent experiments). **n**, TMRM/MTG (Tetramethylrhodamine methylester perchlorate and mitotracker green) ratio as index of mitochondrial membrane potential in control and CerS2$^{\Delta Ins1E}$ cells (*n* = 3 independent experiments). **o**, Successful validation of Pcsk1 antibody specificity (Cell Signaling, #11914, discontinued during our studies) in lysates from control, heterozygous and homozygous Pcsk1$^{\Delta Ins1E}$ cells as well as in Ins1E cells treated with control siRNA and siRNA targeting Pcsk1, as well as control and CerS2$^{\Delta BKO}$ mice. Blot was intentionally overexposed to visualize unspecific band(s). Stain-Free signal was used as loading control. We note that several other commercial antibodies failed this validation in our hands. **p,** Successful validation of Pro-Pcsk1 and Pcsk1 antibody specificity (Cell Signaling #18030) in lysates from control, heterozygous and homozygous Pcsk1$^{\Delta Ins1E}$ cells. Blot was intentionally overexposed to visualize unspecific band(s). Stain-Free signal was used as loading control. Statistical analysis was performed using unpaired two-sided Student's *t*-test with Holm-Sidak correction (b, c, e, g, i-k, p) or unpaired

two-sided Student's *t*-test (d, f, h, l-n). *P*-values are stated in each figure. Data points depict independent experiments (b-m) or individual cells (n). Bar graphs represent means + s.e.m.

Supplementary Figure 3: Additional data for PACS studies.

a, pacSph treatment does not impact on viability of Sgpl1$^{\Delta Ins1E}$ and CerS2:Sgpl1$^{\Delta Ins1E}$ cells. Propidium iodide staining of ethanol (solvent) or pacSph (5µM) treated cells was performed 1h after treatment, followed by flow cytometry (*n* = 3 independent experiments per cell line). **b**, Double cut CrispR/Cas9 knockout strategy for Sgpl1 in Ins1E cells. **c**, Relative mRNA expression of Sgpl1 and various CerS in wildtype Ins1E cells, Sgpl1$^{\Delta Ins1E}$ and CerS2:Sgpl1$^{\Delta Ins1E}$ cells (*n* = 3 independent experiments). Wildtype Ins1E cDNA was also used for qPCR in Extended Data Figure 8b. **d**, Volcano plot showing $\log_2$ fold change of sphingolipids between CerS2:Sgpl1$^{\Delta Ins1E}$ and Sgpl1$^{\Delta Ins1E}$ Ins1E cells plotted against the $-\log_{10}$ *p*-value of a two-sided equal variance *t*-test. $\log_2$ fold change >1 and Benjamini-Hochberg false discovery rate (BH-FDR) <0.05 was used as cut-off for significance. Only lipids detected in all samples were used for calculation. For lipidomic analyses, 4 individual Sgpl1$^{\Delta Ins1E}$ and CerS2:Sgpl1$^{\Delta Ins1E}$ monoclonal cell lines were used, respectively. These 4 cell lines were pooled, respectively, for all pacSph pull-down experiments. **e**, Volcano plot showing $\log_2$ fold change of proteins pulled-down from pacSph-treated (see Fig. 5a) Cers2:Sgpl1$^{\Delta Ins1E}$ (+UV) vs. Sgpl1$^{\Delta Ins1E}$ (-UV) cells plotted against the $-\log_{10}$ p-values of a one-sample two-sided *t*-test against 0. Proteins with $\log_2$ fold change > 1 and a BH-FDR < 0.05 are regarded as SBPs (*n* = 4 independent experiments). **f**, Representative images of pseudoislets 72h after lipofection of trypsinized islet cells with control siRNAs, or a pool of siRNAs targeting Tmed2, followed by gravity-assisted reaggregation ("hanging drop" method). Scale bar: 100 µm. **g-j**, Expression levels of CerS2, CerS5 and CerS6 in human islets after induction of Golgi stress (Brefeldin A; re-analysis of GSE152615, see methods), glucotoxicity (glucose), lipotoxicity (palmitate), or

glucolipotoxicity (glucose+palmitate/Glu+Pal; all three re-analyses of GSE159984, see methods). *n* = 4 (g and h), 5 (i) and 3 (j) independent islet batches from human donors. **k**, Graphical summary. Data points in (a, c) depict individual experiments. Data points in (g-j) represent individual human donors. Bar graphs represent mean ± s.e.m. TPM, transcripts per million.

Supplementary Figure 4: Gating strategies for flow cytometry analyses.

a, Exemplary gating strategy for flow cytometric analyses of beta cell granularity (Extended Data Figure 3i). **b,** Exemplary gating strategy for flow cytometric analyses of EdU incorporation in Ins1E cells (Supplementary Figure 2l). **c,** Exemplary gating strategy for flow cytometric viability analyses of Ins1E cells (Supplementary Figure 3a). FSC-A, forward scatter area; SSC-A side scatter area; FSC-H, forward scatter height; SSC-H, side scatter height; EdU-AF488, 5-ethynyl-2'-deoxyuridine – Alexa Fluor 488; PI-H, propidium iodide height.

Supplementary Figure 1

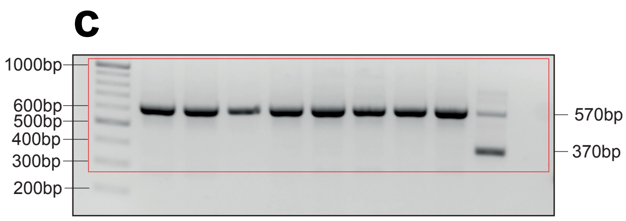

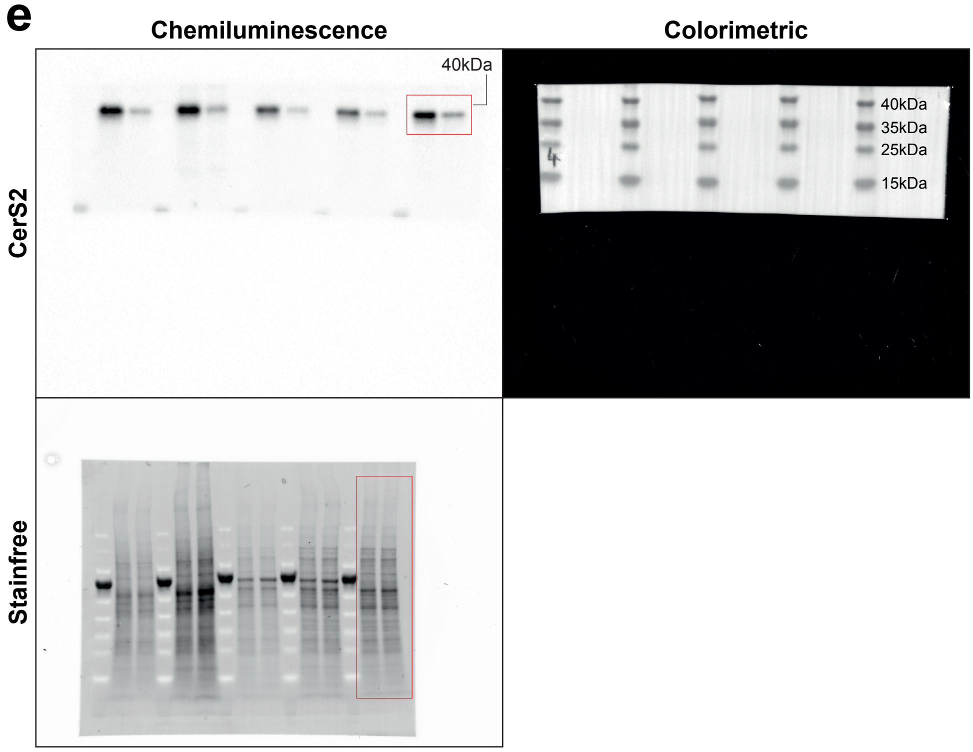

Supplementary Figure 2

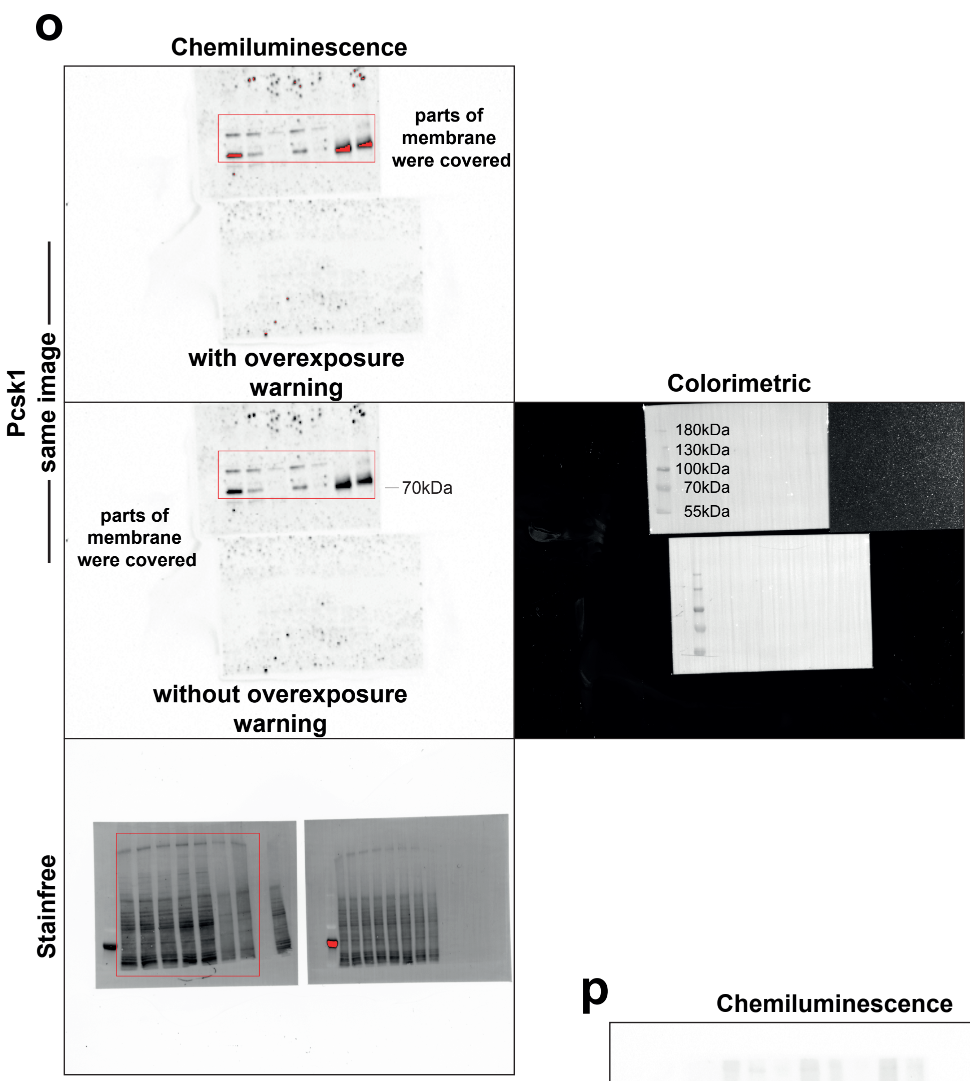

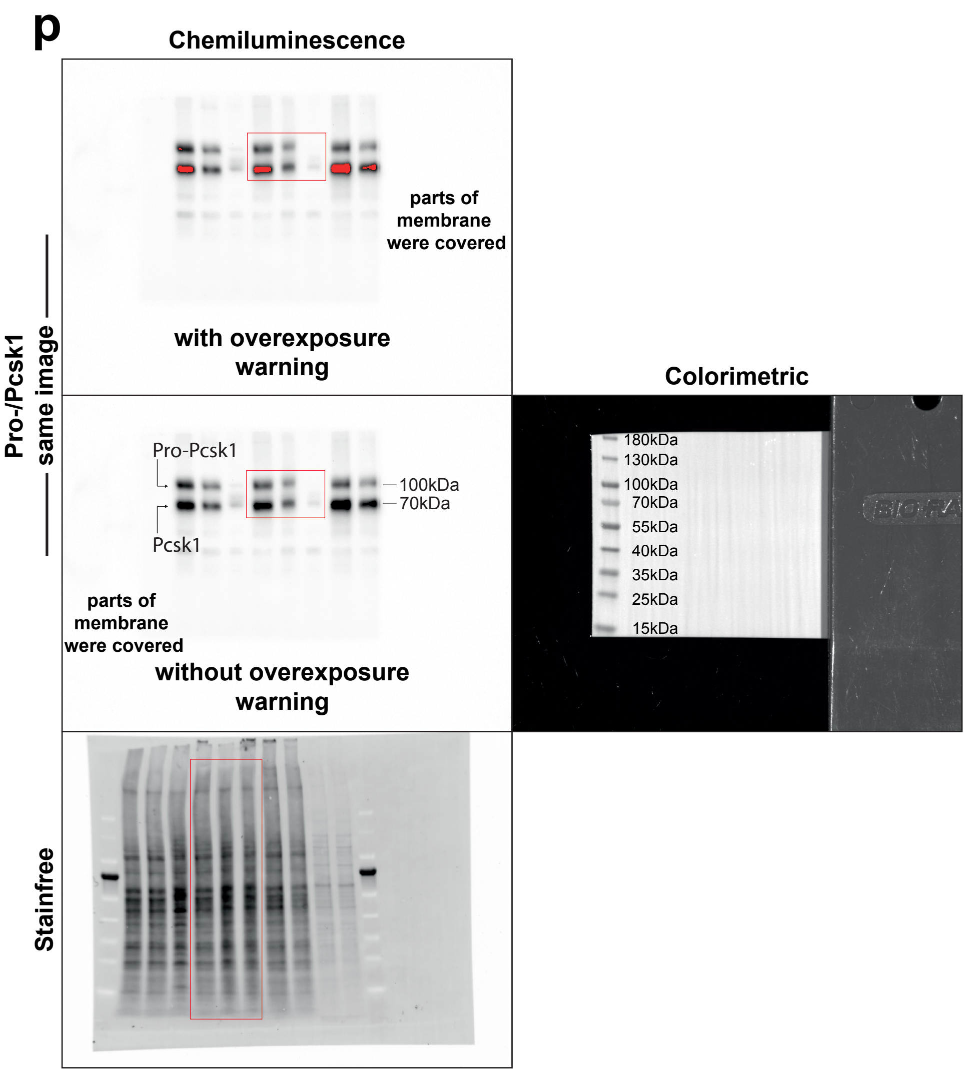

Supplement: Supplementary file 1 — Supplementary Figs. 1–4, figure legends for Supplementary Figs. 1–4 and source blots for Supplementary Figs. 1–4. [file 41556_2022_1027_MOESM1_ESM.pdf]
