# Supplementary material for: Sphingolipid subtypes differentially control proinsulin processing and systemic glucose homeostasis
Source: Nat Cell Biol. 2022 Dec 21;25(1):20–9. doi: 10.1038/s41556-022-01027-2 (PMC9859757; doi:10.1038/s41556-022-01027-2)
Supplement: Supplementary file 2 — Reporting Summary [file 41556_2022_1027_MOESM2_ESM.pdf]

nature portfolio

Corresponding author(s): Belgardt, Bengt-Frederik

Last updated by author(s): 2022/09/23

Reporting Summary

Nature Portfolio wishes to improve the reproducibility of the work that we publish. This form provides structure for consistency and transparency in reporting. For further information on Nature Portfolio policies, see our Editorial Policies and the Editorial Policy Checklist.

Statistics

For all statistical analyses, confirm that the following items are present in the figure legend, table legend, main text, or Methods section.

n/a	Confirmed	
☐	☒	The exact sample size (*n*) for each experimental group/condition, given as a discrete number and unit of measurement
☐	☒	A statement on whether measurements were taken from distinct samples or whether the same sample was measured repeatedly
☐	☒	The statistical test(s) used AND whether they are one- or two-sided<br>*Only common tests should be described solely by name; describe more complex techniques in the Methods section.*
☒	☐	A description of all covariates tested
☐	☒	A description of any assumptions or corrections, such as tests of normality and adjustment for multiple comparisons
☐	☒	A full description of the statistical parameters including central tendency (e.g. means) or other basic estimates (e.g. regression coefficient) AND variation (e.g. standard deviation) or associated estimates of uncertainty (e.g. confidence intervals)
☐	☒	For null hypothesis testing, the test statistic (e.g. *F*, *t*, *r*) with confidence intervals, effect sizes, degrees of freedom and *P* value noted<br>*Give P values as exact values whenever suitable.*
☒	☐	For Bayesian analysis, information on the choice of priors and Markov chain Monte Carlo settings
☒	☐	For hierarchical and complex designs, identification of the appropriate level for tests and full reporting of outcomes
☒	☐	Estimates of effect sizes (e.g. Cohen's *d*, Pearson's *r*), indicating how they were calculated

Our web collection on statistics for biologists contains articles on many of the points above.

Software and code

Policy information about availability of computer code

Data collection	QuantStudio 7 Flex System Software Version 1.3 (Thermo Fisher Scientific)<br>Chemidoc MP System Software/Image lab version 5.2.1 (Biorad)<br>Glomax Discover System Software Version 2.4 (Promega)<br>LSM 880 System Software/Zen Black, various versions (Zeiss)<br>Operetta CLS system software/Harmony Version 4 (PerkinElmer)<br>FACS Calibur CellQuest Pro, various versions (BD Biosciences)<br>FACSAria III system software FACSDiva, various versions (BD Biosciences)<br>Cytoflex S and SRT system software CytExpert, various versions (Beckman Coulter)<br>Xcalibur Software, Orbitrap Tune application, various versions (Thermo Fisher Scientific)<br>Q Exactive System Software, various versions (Thermo Fisher Scientific)<br>Nikon SMZ1270 camera device driver (The Imaging Source, www.theimagingsource.com)<br>Nikon NIS-Elements BR 4.51 (Nikon)<br>TEM system software iTEM Version 5.2 (Olympus Soft Imaging Solution GmbH)<br>Spinning disc microscope system software (Perkin Elmer)<br>Cytation 5 software, Gen5, (BioTek)
Data analysis	Fiji or ImageJ, several versions (https://imagej.net/software/fiji/)<br>Spectronaut Pulsar Version 12.0.20491.5 (Biognosys) and 13.12.200217<br>Proteome Discoverer Version 2.2.0.388 (Thermo Fisher Scientific).<br>Microsoft Excel, various versions (Microsoft)<br>Graphpad Prism Version 7, 8 and 9 (GraphPad)<br>Tracefinder Version 4.1 (Thermo-Fisher)<br>RStudio version 1.2.5001 (RStudio)<br>Perseus Version 1.6.15.0 (MPI of Biochemistry, https://maxquant.net/perseus/)

Image lab, various versions (Biorad)
FlowJo version 10 (BD Biosciences)
R 3.6.3 (https://www.R-project.org/)
CellProfiler version 4.1.3 (Broad Institute, https://cellprofiler.org/)
KNIME version 4.3 (KNIME)
Salmon 1.4.0 (https://combine-lab.github.io/salmon/)
R packages: "DESeq2 1.28.1", dplyr", "ggplot2", "ggrepel", "IHW", "matrixTest"

For manuscripts utilizing custom algorithms or software that are central to the research but not yet described in published literature, software must be made available to editors and reviewers. We strongly encourage code deposition in a community repository (e.g. GitHub). See the Nature Portfolio guidelines for submitting code & software for further information.

Data

Policy information about availability of data

All manuscripts must include a data availability statement. This statement should provide the following information, where applicable:

- Accession codes, unique identifiers, or web links for publicly available datasets
- A description of any restrictions on data availability
- For clinical datasets or third party data, please ensure that the statement adheres to our policy

Ins1E CerS KO MS source data is available at the PRIDE database with the dataset identifiers PXD029848 and PXD029781.
Lipidomics data sets are presented in Supplementary Tables 1-6 and 8.
Analysis of proteomics data of Ins1E KO cells is presented in Supplementary Table 7.
Analysis of beta cell SBPs / SL-protein interactomics (CerS2 dependent and independent) is presented in Supplementary Tables 9-12.
Information on antibodies, primers, sgRNAs and siRNAs is presented in Supplementary Table 13.
Numerical data and uncropped, unprocessed immunoblots for all figures are available as Source Data.
All other data that support the findings of this study are available from the corresponding author upon reasonable request, as mentioned in the manuscript.

Field-specific reporting

Please select the one below that is the best fit for your research. If you are not sure, read the appropriate sections before making your selection.

☒ Life sciences ☐ Behavioural & social sciences ☐ Ecological, evolutionary & environmental sciences

For a reference copy of the document with all sections, see nature.com/documents/nr-reporting-summary-flat.pdf

Life sciences study design

All studies must disclose on these points even when the disclosure is negative.

Sample size	Sample sizes were based on own previous results as well as on published experimental pipelines (PMIDs 32810137 and 25774850). For experiments with animal cohorts, sample sizes were designed to detect a significant and biologically relevant difference between control and Knockout cohorts.
Data exclusions	Data was only excluded when positive controls failed, obvious technical issues occurred or due to euthanization of animals in line with local animal guidelines.
Replication	The number of biological and/or technical replicates as well as independent experiments are reported in each figure legend.
Randomization	Since mice with different genetic manipulations were used, randomization was not applicable. Sex- and age-matched control and Knockout cohorts were run in parallel. For cellular assays, treatments were assigned in a random manner (e.g. it was random, which one out of two Ins1E cell plates was used for +UV-, and which one was used for -UV-treatment).
Blinding	There was no blinding since the individuals planning and performing the experiments and processing and analyzing samples were the same and the treatment groups were known to them.

Reporting for specific materials, systems and methods

We require information from authors about some types of materials, experimental systems and methods used in many studies. Here, indicate whether each material, system or method listed is relevant to your study. If you are not sure if a list item applies to your research, read the appropriate section before selecting a response.

Materials & experimental systems

n/a	Involved in the study
☐	☒ Antibodies
☐	☒ Eukaryotic cell lines
☒	☐ Palaeontology and archaeology
☐	☒ Animals and other organisms
☒	☐ Human research participants
☒	☐ Clinical data
☒	☐ Dual use research of concern

Methods

n/a	Involved in the study
☒	☐ ChIP-seq
☐	☒ Flow cytometry
☒	☐ MRI-based neuroimaging

Antibodies

Antibodies used

Primary antibodies

CerS2 (HPA027262, Sigma-Aldrich)
CerS6 (H00253782-M01, Abnova)
Pcsk1 (PC1) (11914, Cell Signaling, discontinued)
Pcsk1 (PC1) 18030, Cell Signaling)
PDI (ab2792, Abcam)
Tmed2 (sc-376458, Santa Cruz)
TNG46 (MA3-063, Invitrogen)
DDK-Tag (14793, Cell Signaling)
DDK-Tag (8146, Cell Signaling)
anti-FLAG magnetic beads (M8823, Merck)
V5-Tag (13202, Cell Signaling)

Secondary antibodies

Goat Anti-Mouse IgG, H&L Chain Specific Peroxidase Conjugate (401253, Calbiochem)
Goat Anti-Rabbit IgG, H&L Chain Specific Peroxidase Conjugate (401393, Calbiochem)
Donkey anti-Mouse IgG (H+L) Highly Cross-Adsorbed Secondary Antibody, Alexa Fluor 488 (A-21202, Thermo-Fisher)
Donkey anti-Rabbit IgG (H+L) Highly Cross-Adsorbed Secondary Antibody, Alexa Fluor Plus 405 (A48258, Thermo-Fisher)
Donkey anti-Mouse IgG (H+L) Highly Cross-Adsorbed Secondary Antibody, Alexa Fluor Plus 647 (A32787, Thermo-Fisher)
Donkey anti-rabbit IgG (H+L) Highly Cross-Adsorbed Secondary Antibody, Alexa Fluor 647 (A31573, Thermo-Fisher)

Dilutions used for western blot: primary antibodies, 1:1000; secondary antibodies, 1:1000-1:5000.
Dilutions used for immunostaining: primary antibodies, 1:200; secondary antibodies, 1:500.

Validation

The CerS2 antibody was validated in pancreatic islets from beta cell specific CerS2 KO mice as well as in CerS2 Knockout rat Ins1E cells in this study (Supplementary Fig. 1E and Fig. 4A).

The CerS6 antibody was validated using pancreatic islets from beta cell specific CerS6 Knockout mice in parallel experiments, and has been validated with CerS6-deficient mouse tissues before, see PMID 31150623.

The Pcsk1/PC1 antibodies were validated in rat Ins1E Pcsk1 Knockout cells in this study (Supplementary Fig. 2o and 2p).

The Tmed2 antibody was validated in rat Ins1E Tmed2 Knockout cells in this study (Extended Data Fig. 9d).

The PDI and TGN46 antibodies are mouse monoclonal antibodies and were validated by the manufacturers in multiple species and applications.

The antibodies recognizing Peptide-Tags (FLAG/DDK, V5) were validated in western blot and immunofluorescence studies in-house, in addition to validation by the manufacturers.

All secondary antibodies were validated for use in western blot and/or immunofluorescence experiments by overexpressing tagged proteins and omission of primary antibodies in-house, in addition to validation by the manufacturers.

Eukaryotic cell lines

Policy information about cell lines

Cell line source(s)

Ins1E cells (published in Merglen et al., Endocrinology 2004) were used with written permission (Material Transfer Agreement) by the University of Geneva and provided by Prof. Dr. Eckhard Lammert (also co-author of this study).

Authentication

We routinely assessed beta cell identity of the cells by using insulin secretion measurements as well as qPCR/immunoblot/immunofluorescence analyses of beta cell markers such as PDX1, Ins1, Ins2, Nkx6.1. Authentication of CrispR/Cas9 knockouts generated in-house was performed by PCR and qPCR (all cell lines, using rat-specific primers) and immunoblot (CerS2 KO).

Mycoplasma contamination	Ins1E cells were routinely tested negative for Mycoplasma contamination using commercial services (GATC/Eurofins).
Commonly misidentified lines (See ICLAC register)	No commonly misidentified cell lines were used in this manuscript.

Animals and other organisms

Policy information about studies involving animals; ARRIVE guidelines recommended for reporting animal research

Laboratory animals	The following mouse strains were used in this manuscript, as also described in the methods section:<br><br>conditional CerS2: Supplementary Fig. 1b<br>conditional CerS5: PMID 31150623<br>conditional CerS6: PMID 25295788<br>Ins1-Cre: PMID 25500700<br>db/db.BKS: Jackson Laboratories #000642<br>ob/ob.B6: Jackson Laboratories #000632<br>Akita: Jackson Laboratories #003548<br>R26-Tomato: Jackson Laboratories #007909<br>C57BL/6JRj: Janvier #SC-C57J-M<br><br>Information on the number of animals per group as well as sex and age of animals and animal husbandry information is described in each figure legend as well as the methods section.
Wild animals	Wild animals were not involved in the study.
Field-collected samples	No field-collected samples are involved in the study.
Ethics oversight	All animal procedures were approved by the Department for Environment and Consumer Protection of North Rhine-Westphalia, Germany (LANUV). Mice were sacrificed in accordance with the German Animal Protection Law (TSchG) and with approval of the DDZ Institutional Animal Welfare Committee.

Note that full information on the approval of the study protocol must also be provided in the manuscript.

Flow Cytometry

Plots

Confirm that:

☒ The axis labels state the marker and fluorochrome used (e.g. CD4-FITC).

☒ The axis scales are clearly visible. Include numbers along axes only for bottom left plot of group (a 'group' is an analysis of identical markers).

☒ All plots are contour plots with outliers or pseudocolor plots.

☒ A numerical value for number of cells or percentage (with statistics) is provided.

Methodology

Sample preparation	No rare cell population was isolated or complex flow cytometry protocol was used in this study.<br><br>Three studies using flow cytometry are presented in this manuscript with results, and all are standard flow cytometry experiments. For determination of proliferation of wildtype and CerS2 deficient Ins1E cells, we used a commercial EdU incorporation kit (Click-iT™ EdU Alexa Fluor™ 488 Flow Cytometry Assay Kit, C10425, Thermo Fisher). For determination of any potential influence of pacSph on Sgpl1 KO and CerS2:Sgpl1 DKO Ins1E cell viability, we used propidium iodide staining after pacSph treatment according to published protocols (cited in this manuscript). For quantification of beta cell granularity, we used side scatter-area (SSC-A) analysis of reporter mice.<br><br>For singularization of CrispR-plasmid transfected Ins1E cells, we used green and red fluorescent proteins encoded by the CrispR-plasmids to sort Ins1E cells into 96 well plates, as described in the methods part.
Instrument	FACS Calibur (BD Biosciences) for Flow cytometry, FACS Aria III (BD Biosciences) and Cytoflex SRT (Beckman Coulter) for singularization of CrispR plasmid transfected cells, Cytoflex S (Beckman Coulter) for SSC-A analysis.
Software	FlowJo Version 10 (BD Biosciences).
Cell population abundance	No quantitative sorting was used, no rare cell populations were investigated.

Gating strategy

We used cells with no EdU treatment as negative control for EdU-staining, unstained cells + boiled cells as negative and positive controls for PI staining, and glibenclamide treated islets as positive control for SSC-A measurements. Gating strategy is shown in Supplementary Fig. 4.

☒ Tick this box to confirm that a figure exemplifying the gating strategy is provided in the Supplementary Information.
