# Supplementary figures and images for: Sphingolipid subtypes differentially control proinsulin processing and systemic glucose homeostasis
Source: Nat Cell Biol. 2022 Dec 21;25(1):20–9. doi: 10.1038/s41556-022-01027-2 (PMC9859757; doi:10.1038/s41556-022-01027-2)

Figure 4

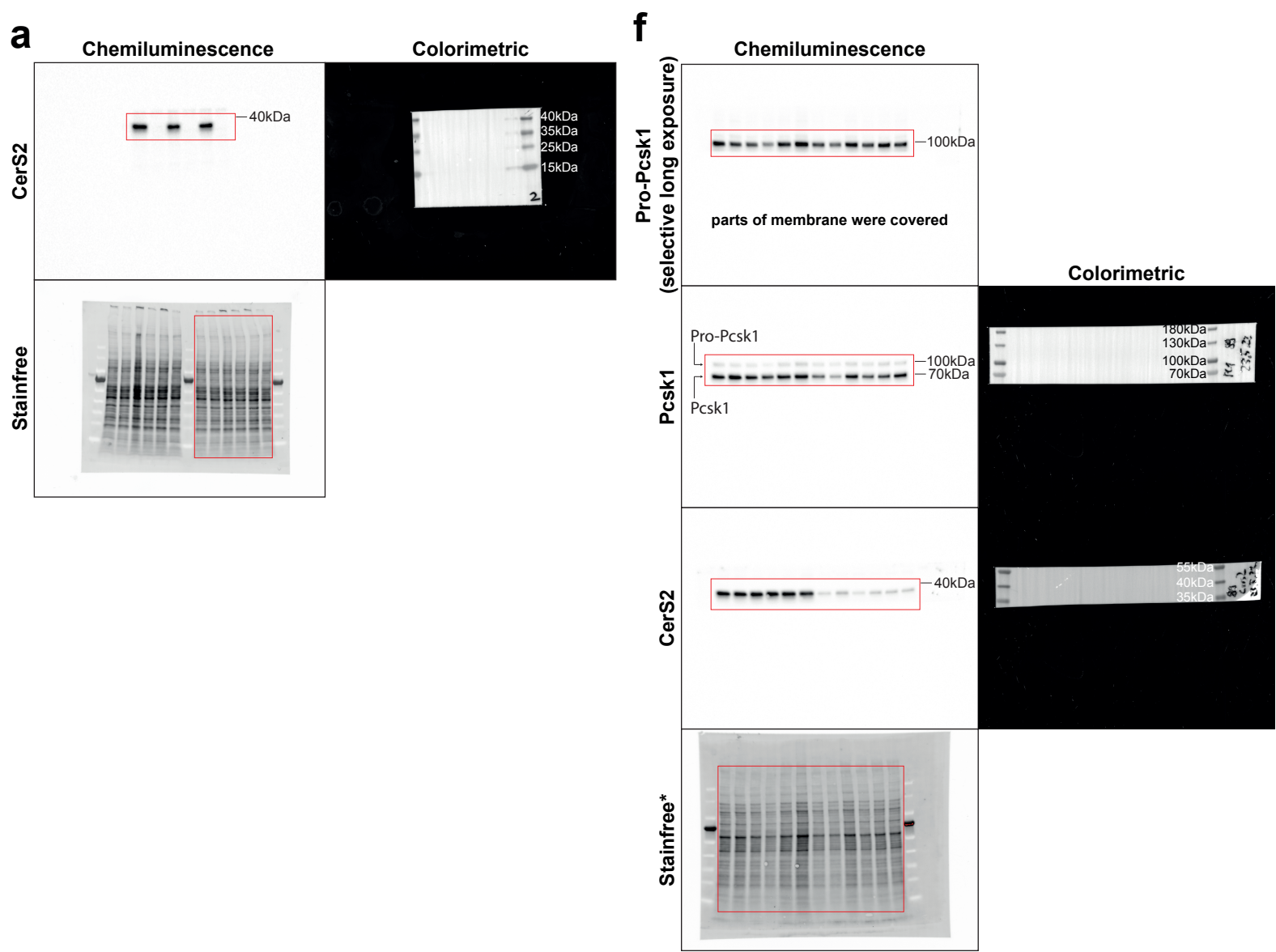

\*Stainfree image is the same as in Ext. Data Figure 7e

Supplement: Source Data Fig. 4 — Unprocessed western blots. [file 41556_2022_1027_MOESM9_ESM.pdf]

Figure 5

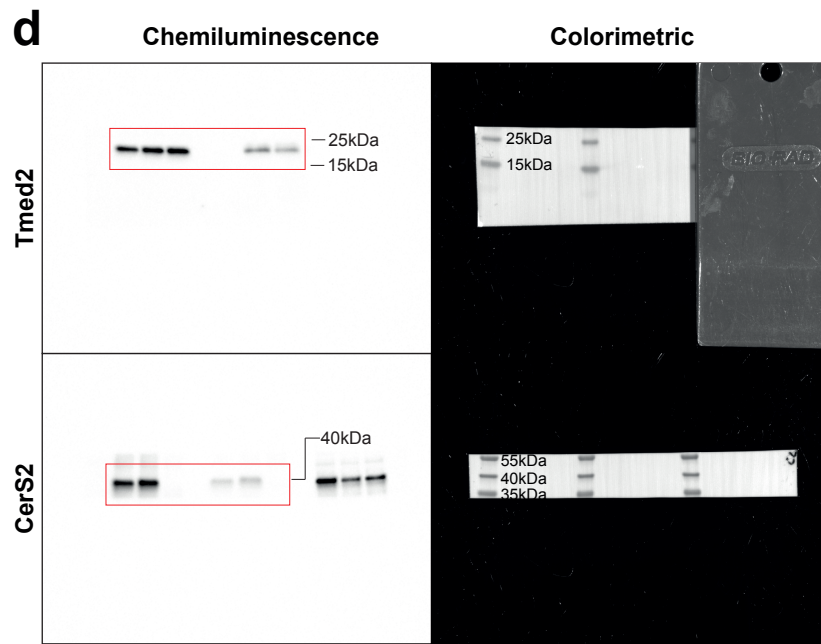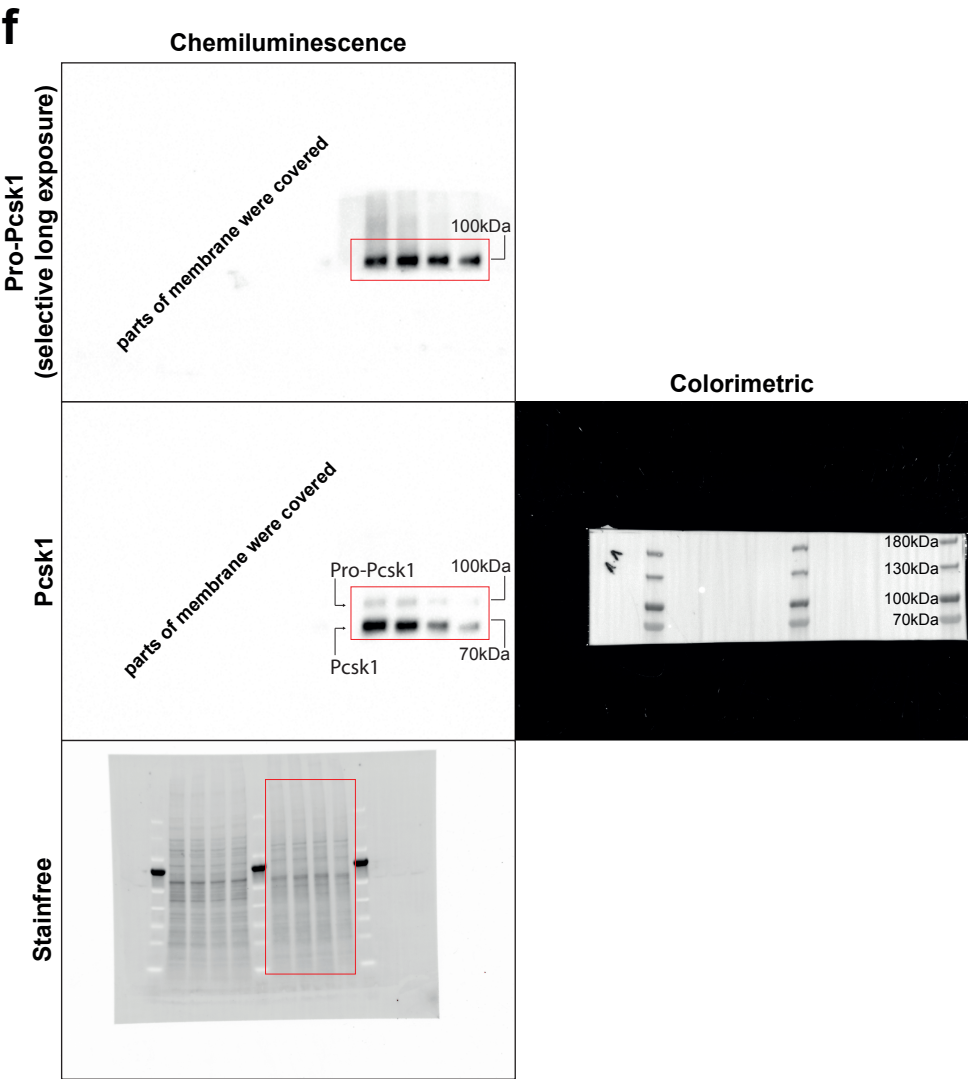

Supplement: Source Data Fig. 5 — Unprocessed western blots. [file 41556_2022_1027_MOESM11_ESM.pdf]

Extended Data Figure 4

**a**

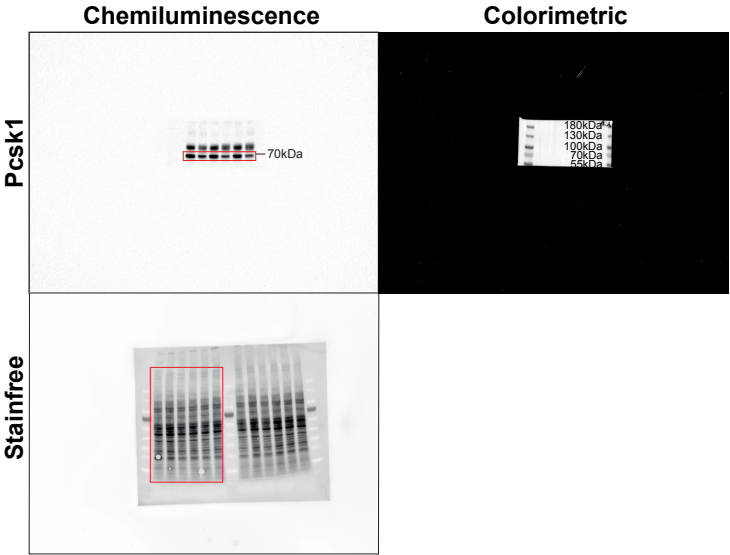

**b**

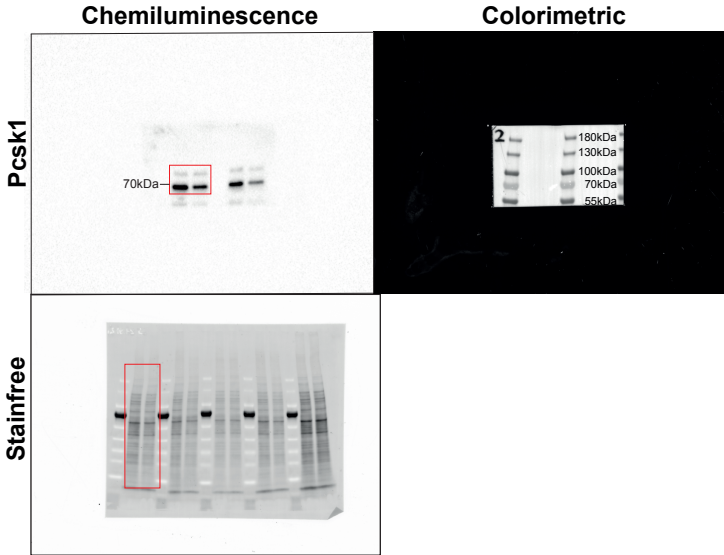

**c**

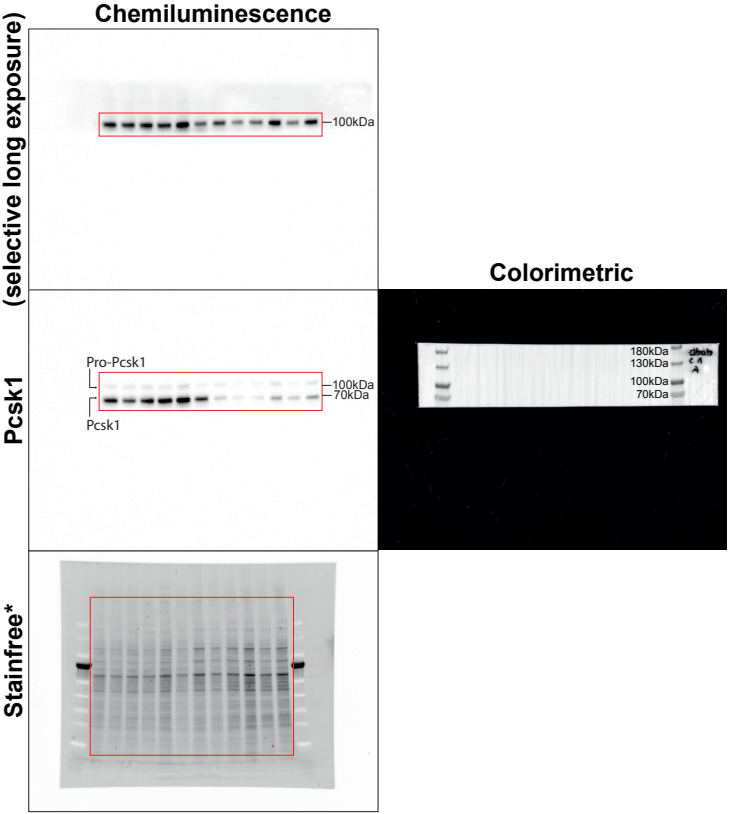

**e**

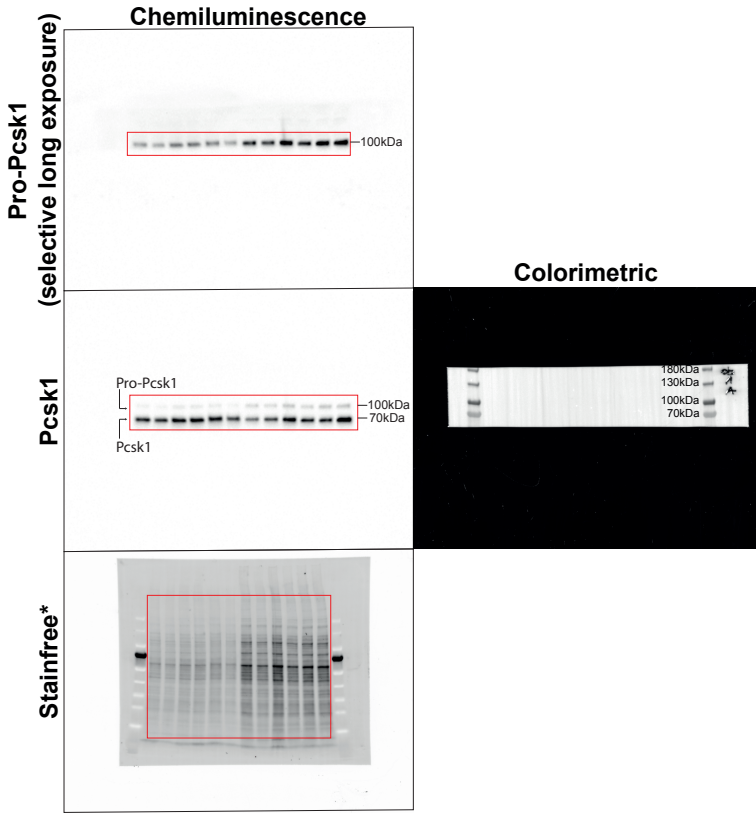

\*Stainfree images are the same as in Ext. Data Figure 7f and g

Supplement: Source Data Extended Data Fig. 4 — Unprocessed western blots. [file 41556_2022_1027_MOESM16_ESM.pdf]

Extended Data Figure 5

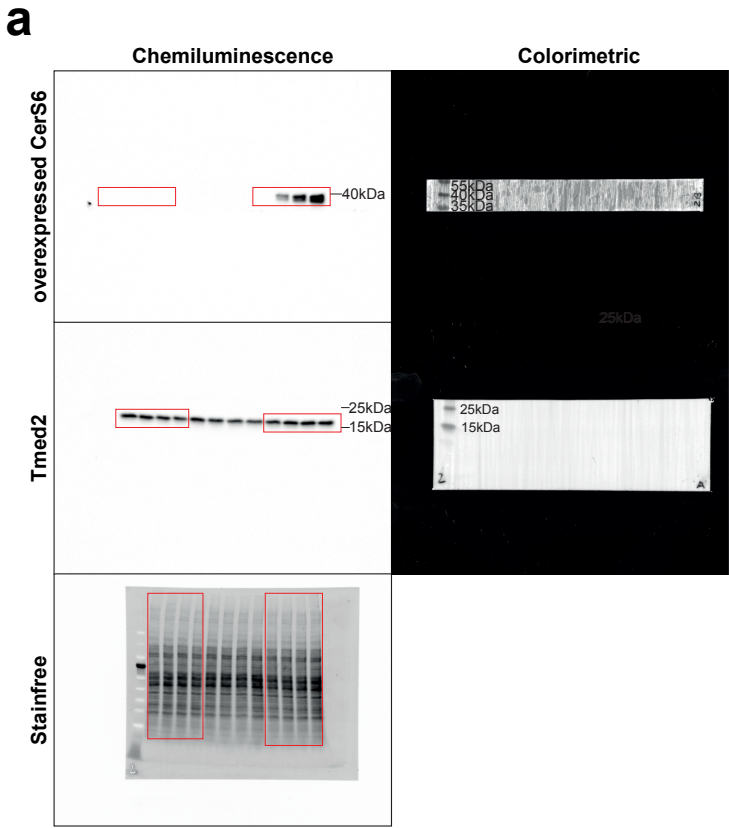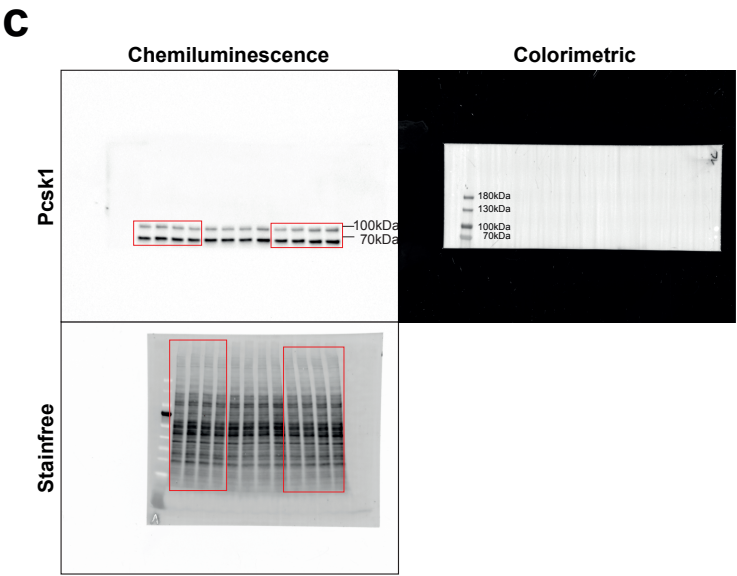

Supplement: Source Data Extended Data Fig. 5 — Unprocessed western blots. [file 41556_2022_1027_MOESM18_ESM.pdf]

Extended Data Figure 8

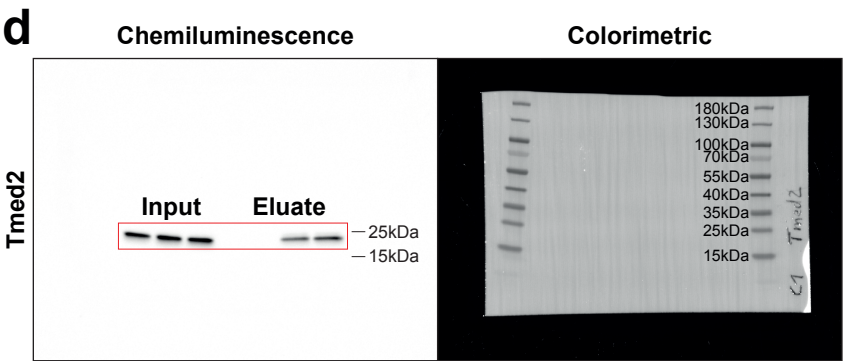

Supplement: Source Data Extended Data Fig. 8 — Unprocessed western blot. [file 41556_2022_1027_MOESM24_ESM.pdf]

Extended Data Figure 9

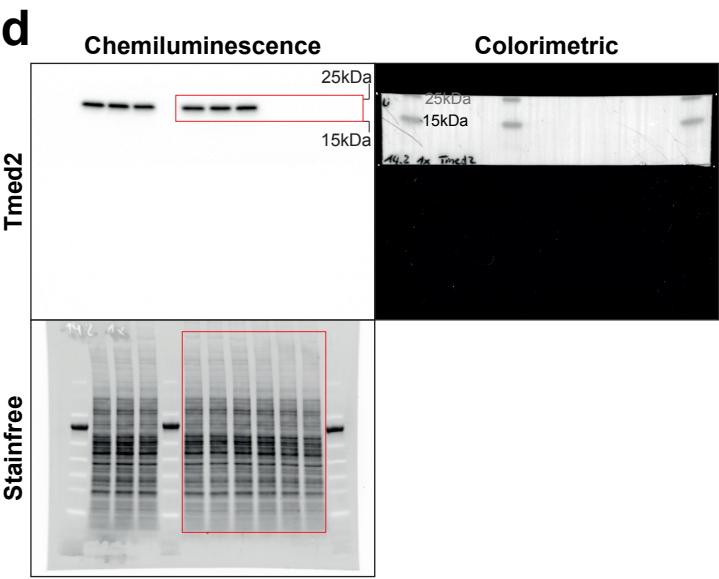

Supplement: Source Data Extended Data Fig. 9 — Unprocessed western blot. [file 41556_2022_1027_MOESM26_ESM.pdf]

Extended Data Figure 10

**a**

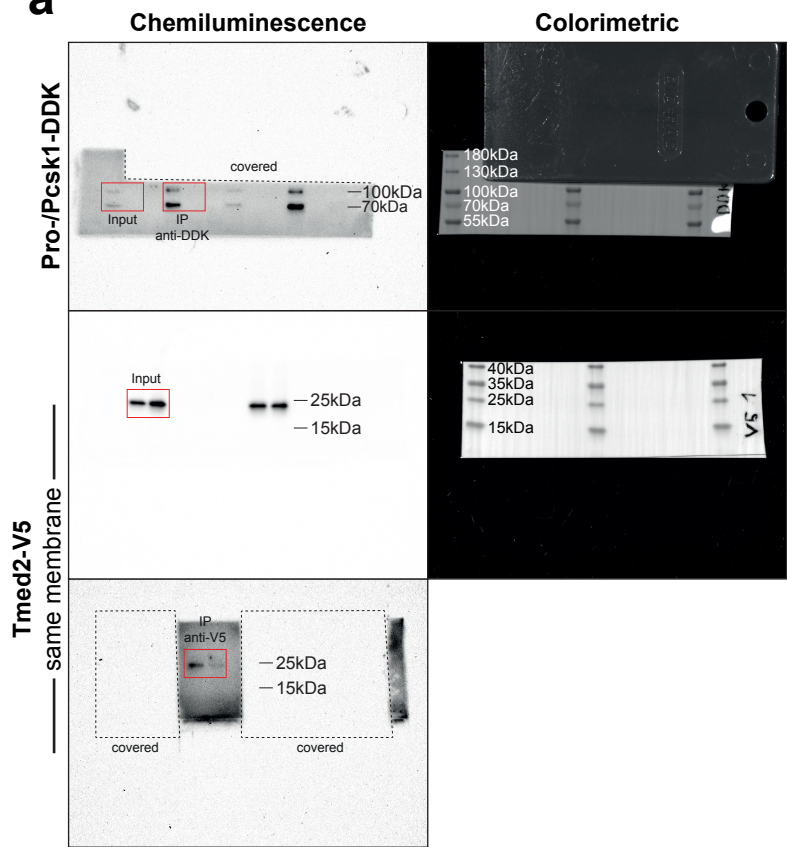

Supplement: Source Data Extended Data Fig. 10 — Unprocessed western blots. [file 41556_2022_1027_MOESM28_ESM.pdf]
