# Supplementary material for: Sphingolipid subtypes differentially control proinsulin processing and systemic glucose homeostasis
Source: Nat Cell Biol. 2022 Dec 21;25(1):20–9. doi: 10.1038/s41556-022-01027-2 (PMC9859757; doi:10.1038/s41556-022-01027-2)
Supplement: Source Data Extended Data Fig. 6 — Unprocessed TLC image. [file 41556_2022_1027_MOESM20_ESM.pdf]

Extended Data Figure 6

a

Completely unprocessed

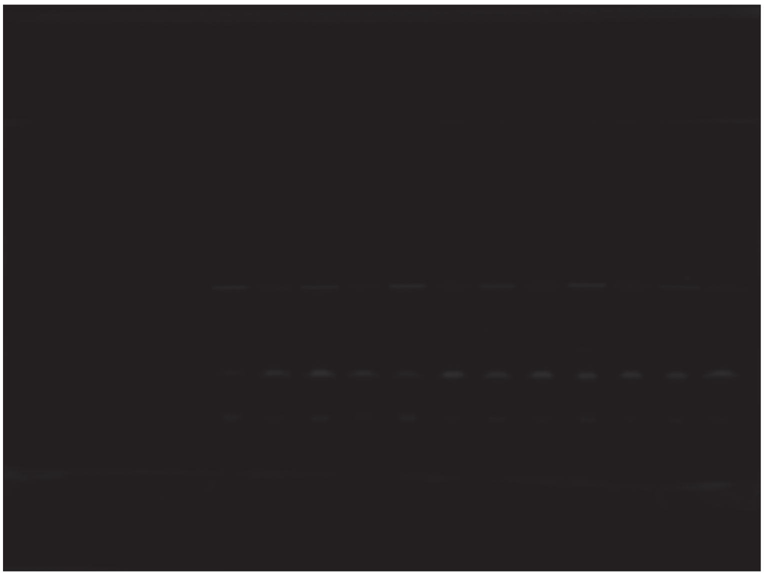

Cropped and inverted*

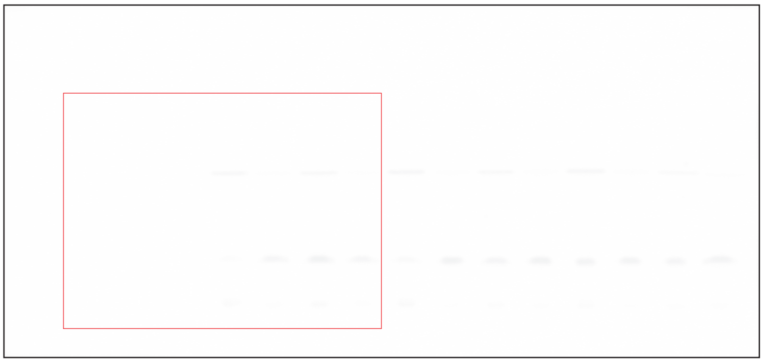

***For Ext. Data Figure 6a, background substraction was performed additionally**
